# Supplementary material for: Polyamide-based membranes with structural homogeneity for ultrafast molecular sieving
Source: Nat Commun. 2022 Jan 25;13:500. doi: 10.1038/s41467-022-28183-1 (PMC8789816; doi:10.1038/s41467-022-28183-1)
Supplement: Supplementary file 1 — Supplementary Information [file 41467_2022_28183_MOESM1_ESM.pdf]

Supporting Information

for

Polyamide-based membranes with structural homogeneity for ultrafast molecular sieving

Liang Shen [a,b,1], Ruihuan Cheng [c,1], Ming Yi [a,b,1], Wei-Song Hung [d,e], Susilo Japip [f], Lian Tian [a,b], Xuan Zhang [a,b], Shudong Jiang [g], Song Li [c,*] and Yan Wang [a,b,*]

[a] Key Laboratory of Material Chemistry for Energy Conversion and Storage (Huazhong University of Science and Technology), Ministry of Education, Wuhan, 430074, P.R. China

[b] Hubei Key Laboratory of Material Chemistry and Service Failure, School of Chemistry and Chemical Engineering, Huazhong University of Science & Technology, Wuhan, 430074, P. R. China

[c] School of Energy and Power Engineering, Huazhong University of Science & Technology, Wuhan, 430074, P. R. China

[d] Graduate Institute of Applied Science and Technology, National Taiwan University of Science and Technology, Taipei, 10607, Taiwan

[e] R&D Centre for Membrane Technology, Chung Yuan Christian University, Taoyuan, 32023, Taiwan

[f] Department of Chemical & Biomolecular Engineering, National University of Singapore, 10 Kent Ridge Crescent, Singapore 119260, Singapore

[g] College of Chemistry and Chemical Engineering, Anhui University, 111 Jiulong Road, Hefei, Anhui, 230601, P.R. China

[1] these authors contributed equally to this work

* Corresponding authors: songli@hust.edu.cn (Song Li)

wangyan@hust.edu.cn (Yan Wang)

List of Content:

Supplementary Methods

Supplementary Figures S1-S21

Supplementary Tables S1- S12

Supplementary References 1-77

1. Supplementary Methods

1.1 PALS characterization of as-fabricated TFC membranes

The detailed experiment procedure for PALS characterization is described below. A variable mono energetic slow positron beam was operated in the range of 0–30 keV positron incident energy (equivalent to a mean depth of 0−10 μm) at room temperature under a vacuum of ~10^{-8} torr. The positron source is of a 50 mCi of ^{22}Na radioisotope beam. Each membrane sample for PALS characterization was in area of 1*1 cm^2. The as-fabricated membrane samples were measured by two positron annihilation techniques, including Doppler broadening of energy spectra (DBES) and positron annihilation lifetime (PAL) measurements. The DBES spectra were measured using a solid state HP Ge detector (EG & G Ortec) at a counting rate of approximately 3000 counts per second (cps). The total number of counts for each DBES was 1.0 million. The S parameter data from DBES was fitted by VEPFIT program. The PALS spectra were obtained by taking coincident events between two signals—the start signal detected by a multichannel plate from the secondary electrons and the stop signal discerned by a BaF_2 lifetime detector from the annihilation photons at a counting rate of ~200-300 cps. A PALS spectrum contains 2.0 million counts. The positron lifetimes (t) and intensities (I) were determined by PATFIT program, and the lifetime distribution was obtained by MELT analysis.

2. Supplementary Figures

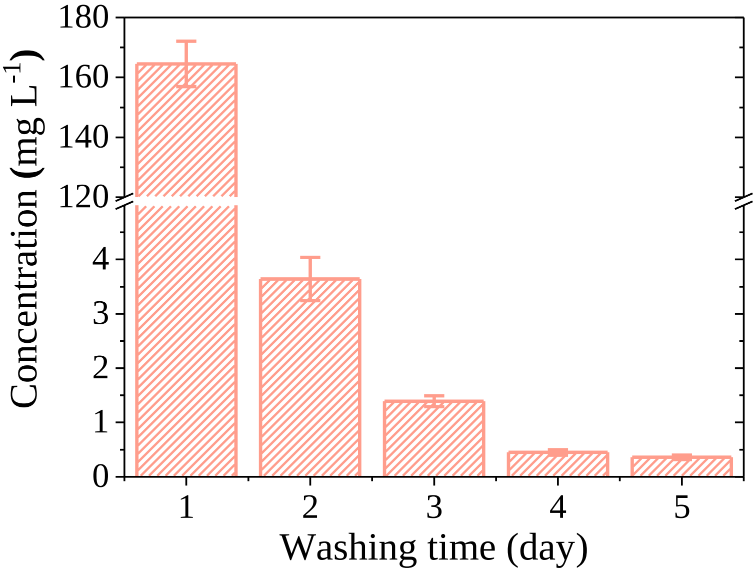

Supplementary Figure 1. NaCl concentration in the washing water of the MPD@NaCl membrane. The error bars represent the standard deviation and were calculated on the basis of at least three data points measured from different samples. It can be seen that NaCl concentration in the washing water sharply decreases after the first day immersion and gradually slows down in the following days. It can be deduced that a large amount of salt removed in the first day is generally the salt trapped in the substrate membrane as well as between the substrate membrane and the PA layer. However, after the first-day washing, the salt trapped in the PA layer can be partially washed out with a longer washing duration (5 days) due to the complexion between the salt ions and the carbonyl groups of the PA network[15-17,23,24].

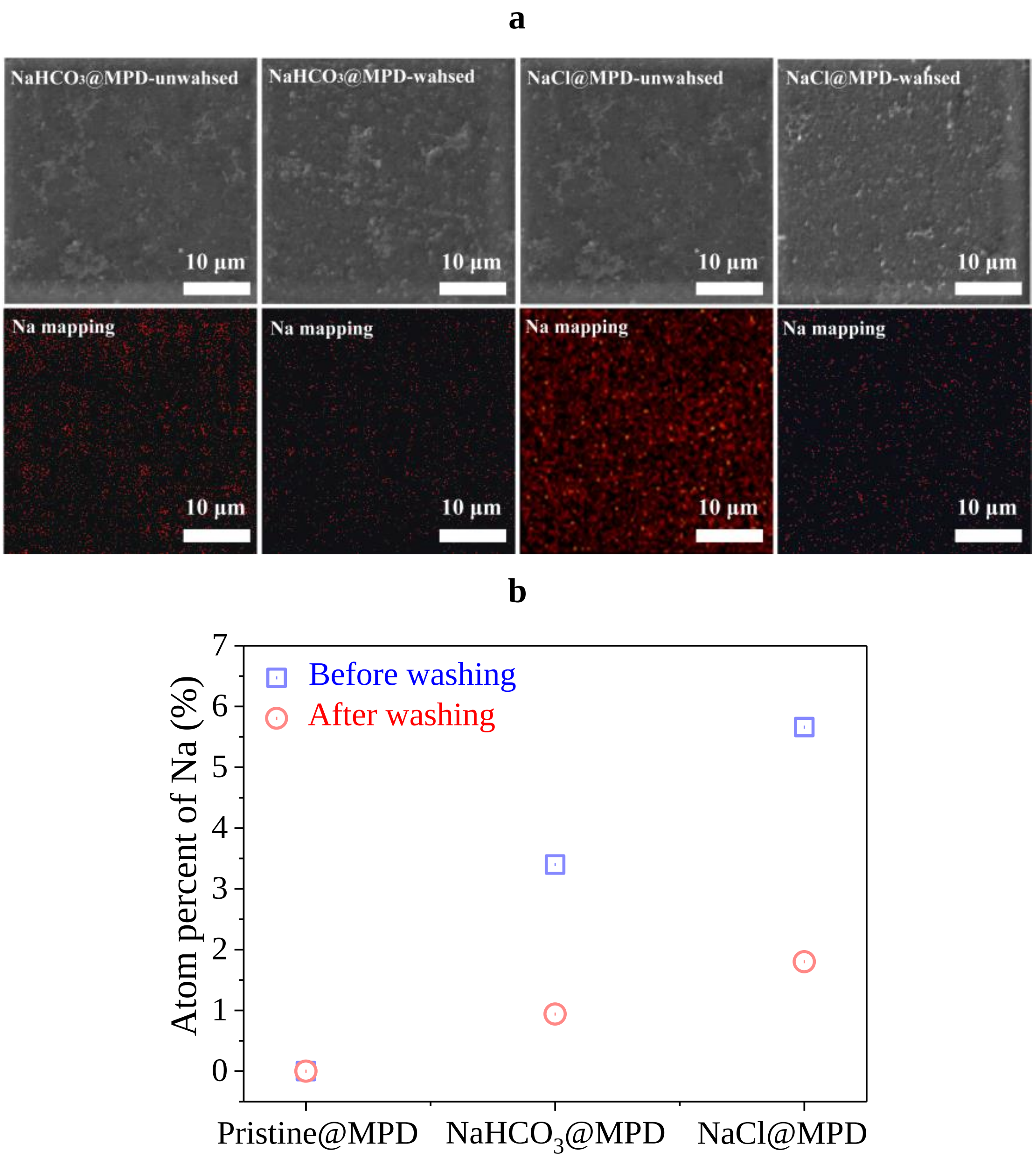

Supplementary Figure 2. EDX characterization results. (a) (top) SEM images and (bottom) corresponding Na mapping images of modified membranes before and after washing; **(b)** atom percent of Na in the pristine and modified membranes before and after washing as examined by EDX analysis. It can be found that Na atom percentage in modified PA layers sharply decreases after washing.

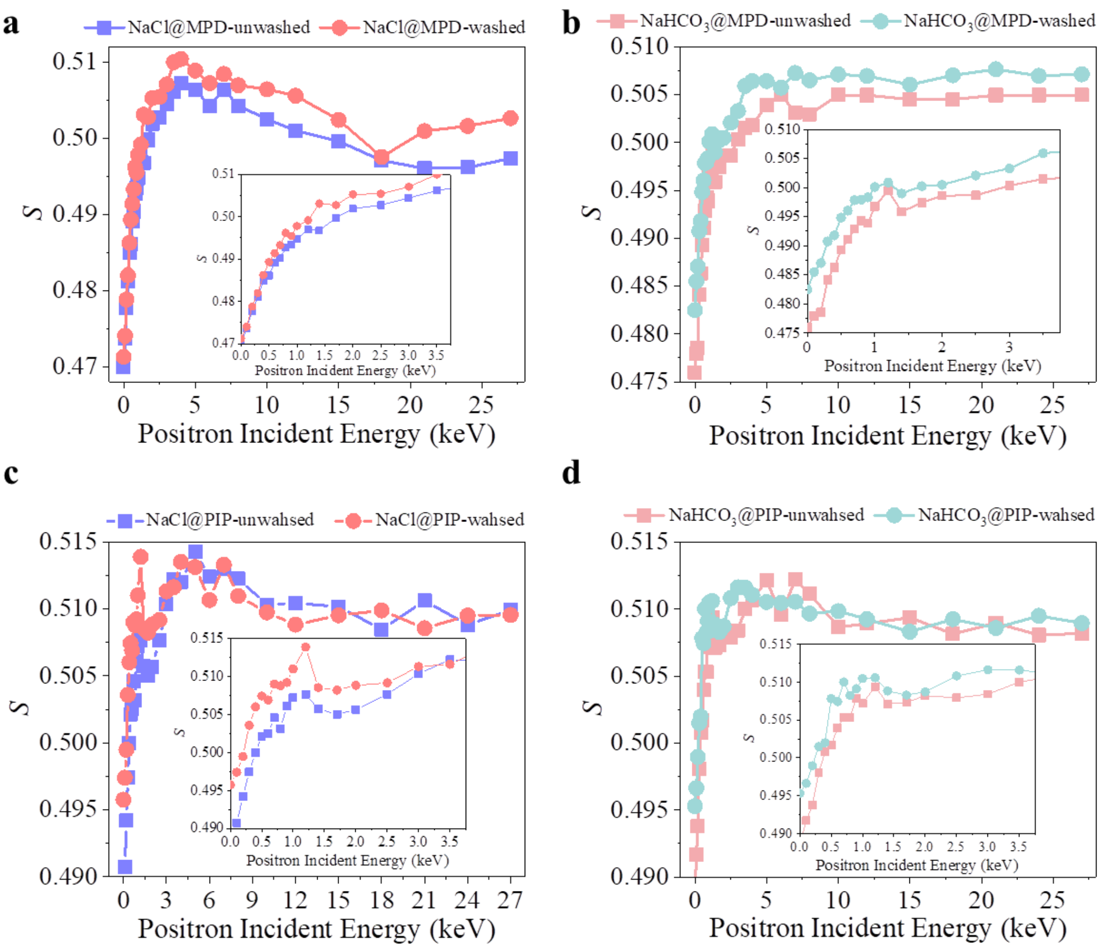

Supplementary Figure 3. PAS results of modified membranes before and after washing. (a) NaCl@MPD, **(b)** $NaHCO_3$@MPD, **(c)** NaCl@PIP, and **(d)** $NaHCO_3$@PIP membranes. *S* profiles as a function of positron incident energy of salt-modified membranes before and after washing. It can be seen that *S* values of salt-modified membranes increase after washing, indicating the increased free volume. The increased free volume of NaCl-modified membrane is solely resulted from the removal of trapped NaCl salts. Instead, the increased free volume of $NaHCO_3$-modified membrane is contributed by both the removal of trapped inorganic salts and generated CO_2 nanobubbles by the reaction between $NaHCO_3$ and IP byproduct HCl. However, the later factor is believed to outweigh the former factor. $NaHCO_3$ in the reaction zone would be consumed by the byproduct HCl generating CO_2 as shown in Supplementary Equation 1. It can be seen that 1 mole $NaHCO_3$

consumed will generate 1 mole NaCl and 1 mole CO_2. Supplementary Figures 3b and 3d show that *S* values of $NaHCO_3$-modified membranes also increase after washing, indicating the increased free volume resulted from the partial removal of generated NaCl. However, the trapped salt in the PA layer can be only partially removed according to the AAS result (< 3%). Meanwhile, all generated CO_2 can create nanobubbles in the PA layer. Therefore, the increased free volume of $NaHCO_3$-modified membranes should be predominantly contributed by the nanobubbles of CO_2 generated.

$$NaHCO_3 + HCl = NaCl + H_2O + CO_2 \uparrow \quad (1)$$

a

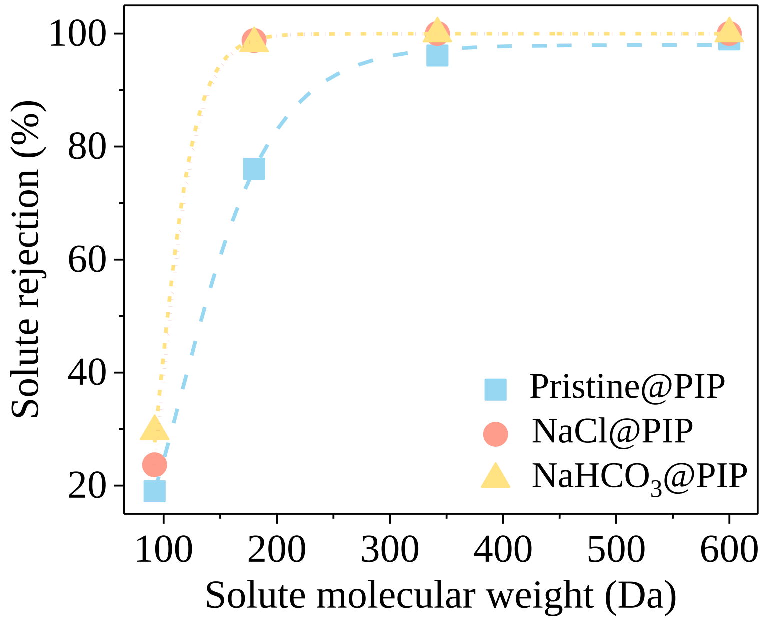

b

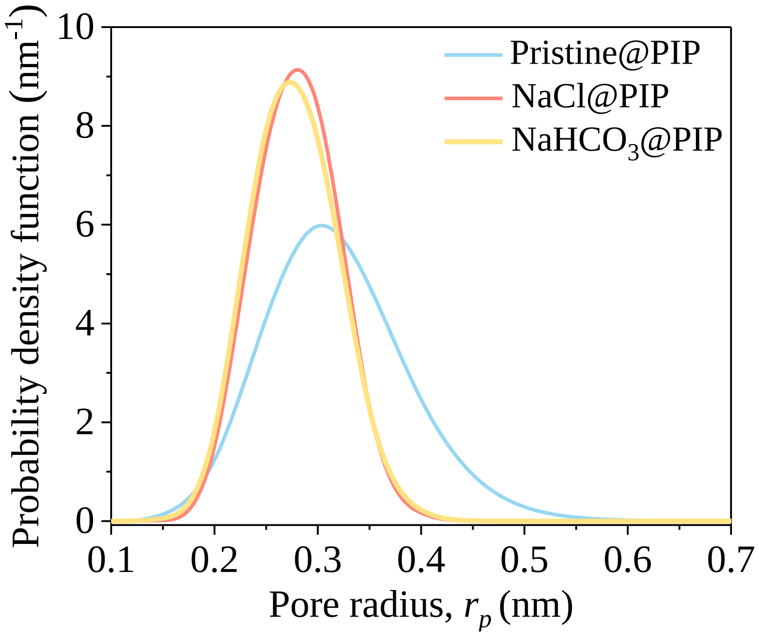

Supplementary Figure 4. Rejection test results of neutral organic solutes. (a) Rejection of neutral organic solutes with different molecular weights by the pristine salt-modified TFC membranes formed by PIP/TMC. **(b)** Pore size distribution of the pristine salt-modified TFC membranes formed by PIP/TMC.

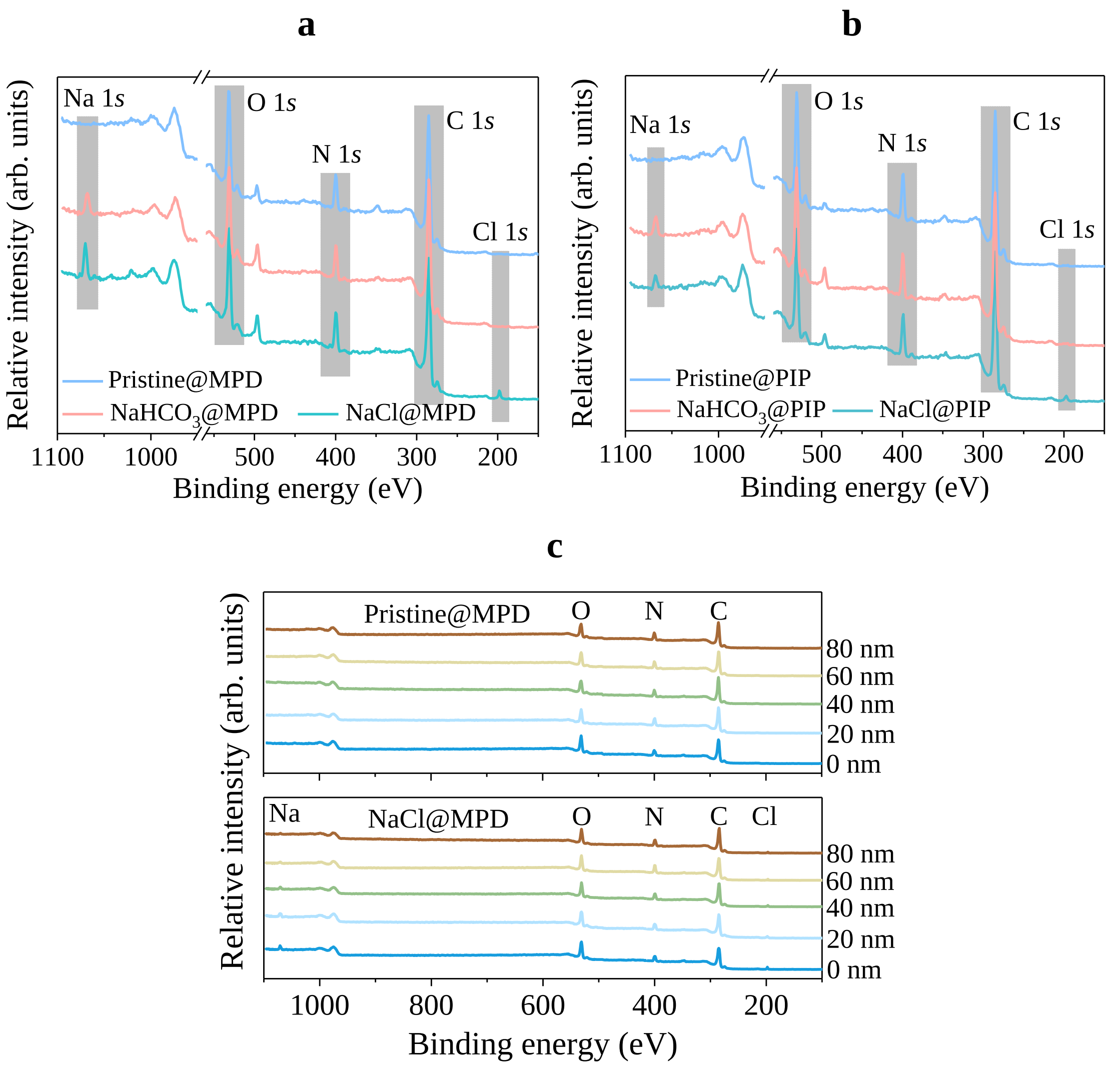

Supplementary Figure 5. XPS characterization results. Wide-scan XPS spectra of the pristine and modified membranes. Membranes formed by **(a)** MPD and TMC, and **(b)** PIP and TMC. **(c)** In-depth profiles of membranes Pristine@MPD and NaCl@MPD. It can be found that peaks representing C, O, N atoms appear in all curves. Additionally, the peak of Na element is found in curves of modified membranes, and the peak of Cl element is noticed in the curve of NaCl-modified membrane. Meanwhile, Na content in NaCl@MPD membrane decreases with the deeper detection depth of the PA layer.

a

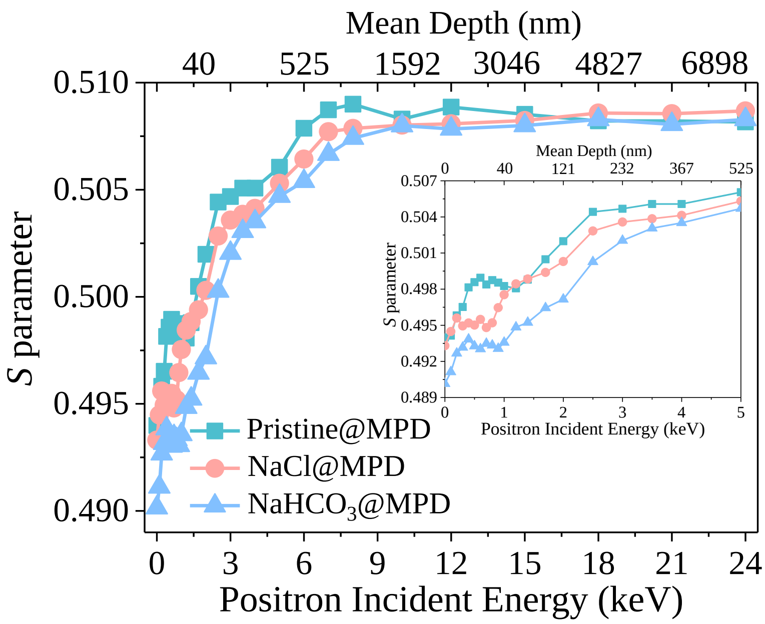

b

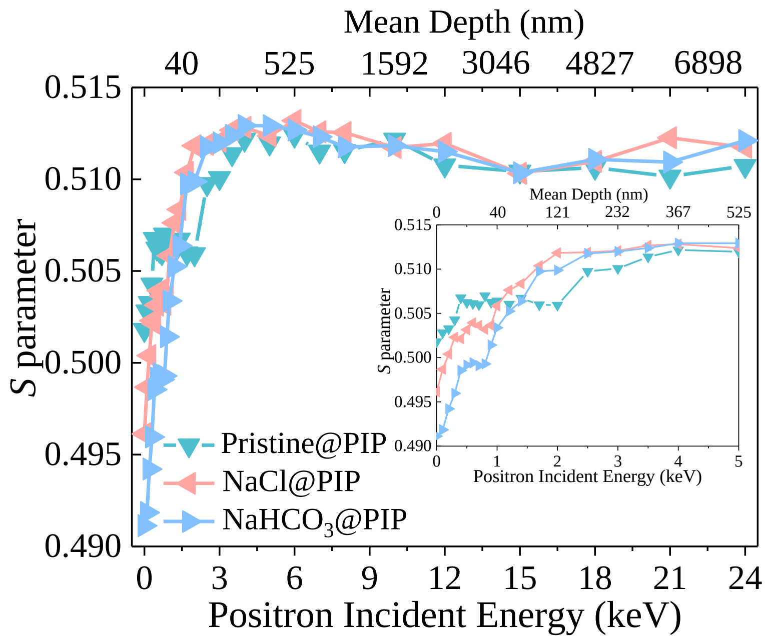

Supplementary Figure 6. PAS results of the pristine and modified membranes. *S* profiles of the pristine and modified membranes as a function of the positron incident energy for **(a)** MPD/TMC and **(b)** PIP/TMC systems. Supplementary Figure 6 shows the *S* profiles of the pristine and modified membranes as a function of positron incident energy (PIE). It can be observed that all profiles present the similar variation

trend to PIE. In this study, the PIE ranges from 0.5 to 3 keV representing the PA selective layer. It can be found in Supplementary Figure 6a that *S* values of modified membranes are lower than that of the pristine membrane, particularly for the $NaHCO_3$@MPD membrane, indicating the formation of a denser PA layer with small free volume. Similarly, the *S* values of NaCl@PIP and $NaHCO_3$@PIP membranes are also lower than that of Pristine@PIP membrane as shown in Supplementary Figure 6b. The results suggest that the addition of inorganic salts does favor the formation of a dense PA layer.

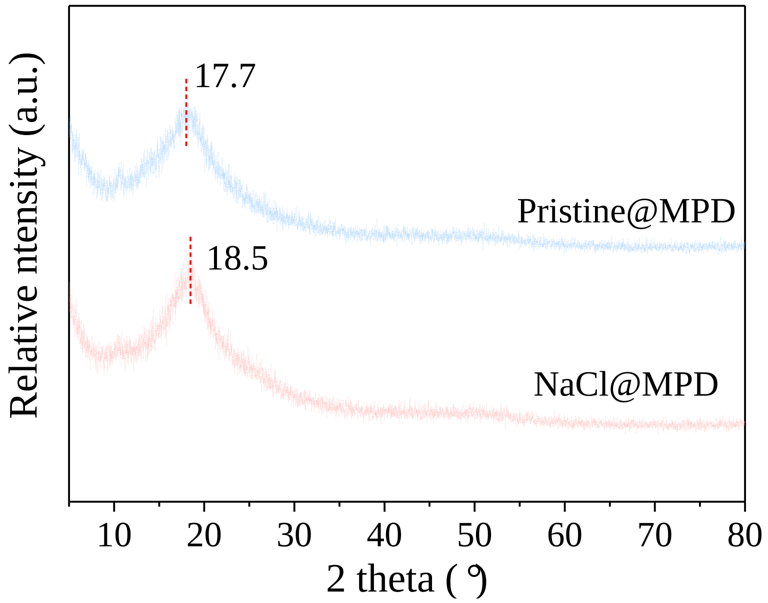

Supplementary Figure 7. XRD patterns of the pristine and NaCl-modified TFC membranes. It can be seen that the characteristic peak at 2θ = 17.7 ° representing the polyamide in the curve of Pristine@MPD membrane shifts to 18.5 ° in that of NaCl@MPD membrane, suggesting the smaller *d*-spacing of modified PA layer.

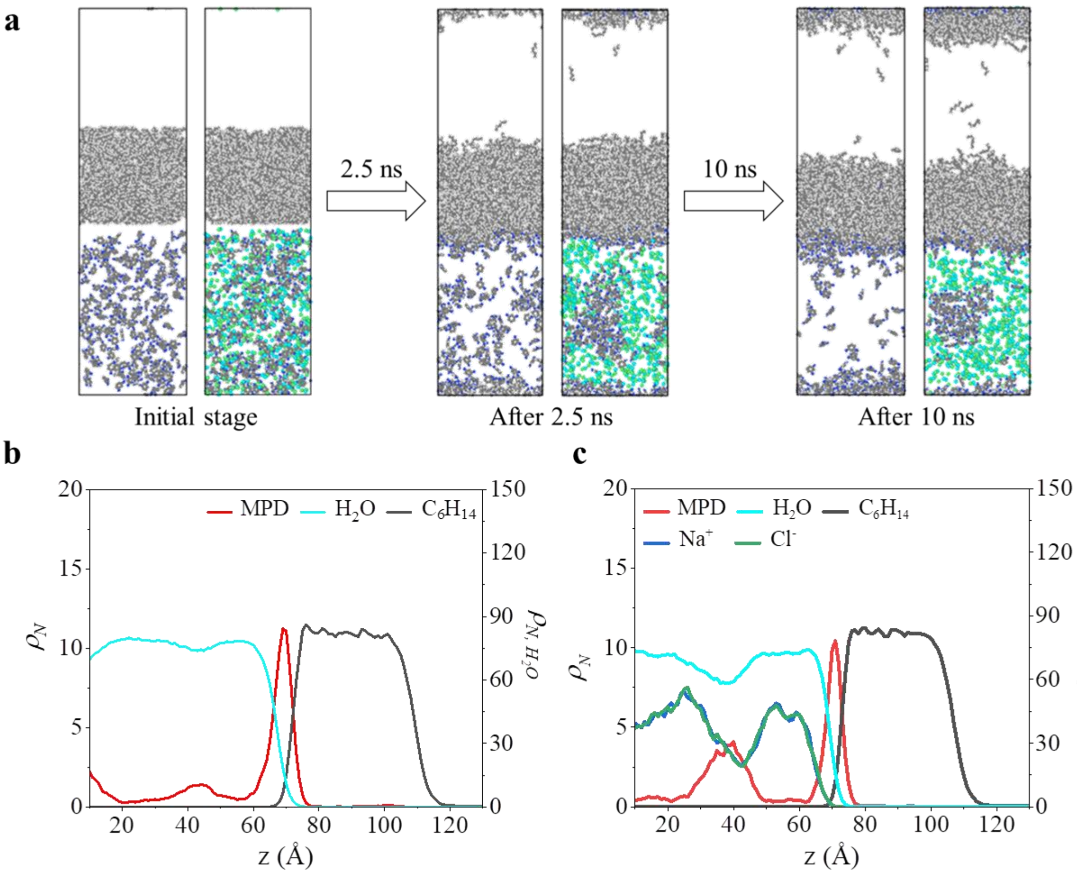

Supplementary Figure 8. MD simulation results. (a) The snapshots describe the diffusion behaviors of MPD molecules in the system with (right) and without (left) NaCl, respectively. It can be clearly found that ions tend to accumulate and surround MPD molecules gradually near the water-oil interface. In contrast, more MPD molecules are inclined to assemble at the interface without NaCl. **(b)** The number density (ρ_N) distribution of MPD (red line), H_2O (cyan line) and hexane (black line) along *z* direction in the system without NaCl. **(c)** The number density (ρ_N) distribution of MPD (red line), H_2O (cyan line), hexane (black line) Na^+ (blue line), and Cl^- (green line) ions along *z* direction in the system with NaCl. The water/hexane interface width of the two MD systems was ~70 Å according to the distribution of water and hexane molecules (Supplementary Figures 8b and 8c).

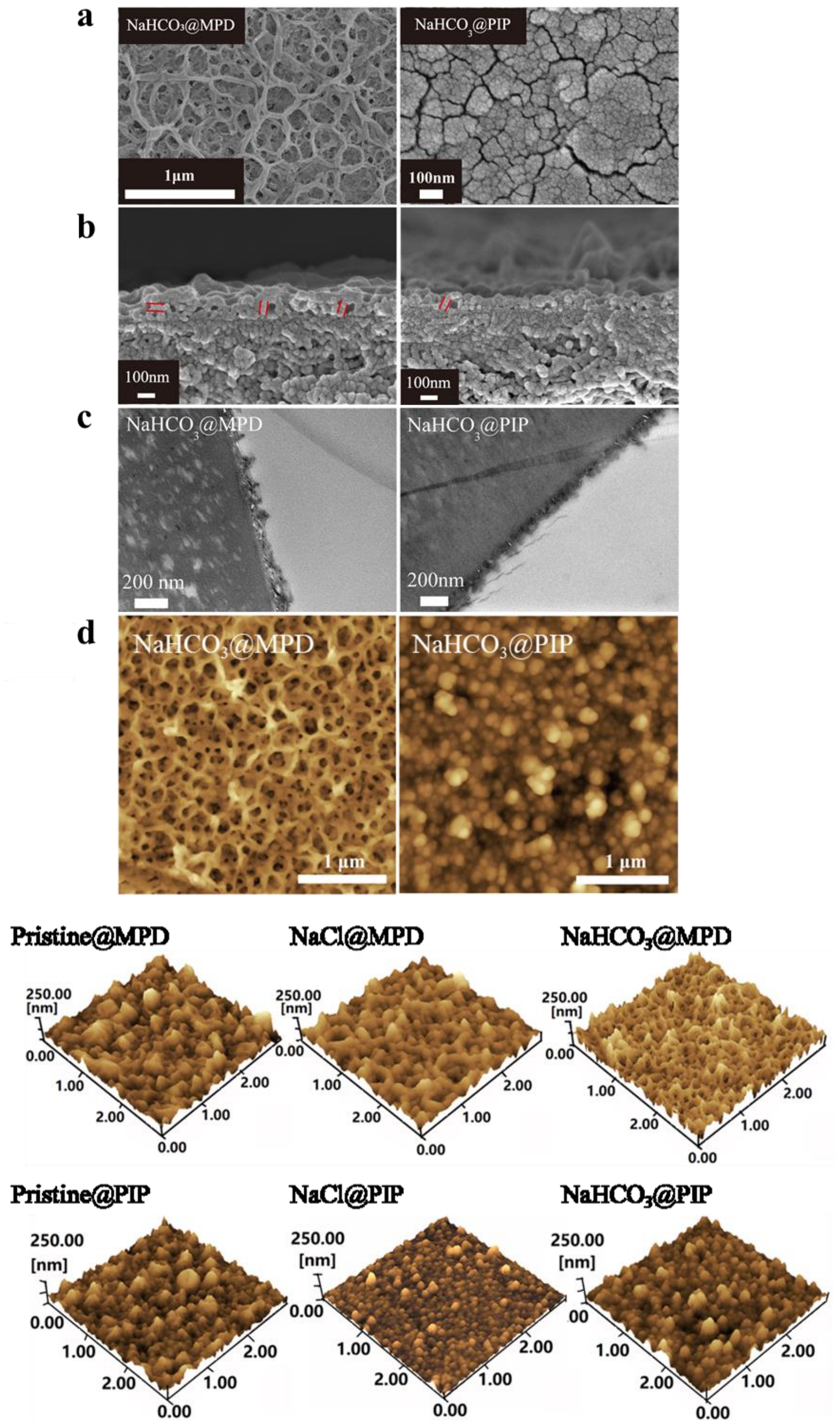

Supplementary Figure 9. Morphology properties of the pristine and modified membranes. **(a)** Surface SEM images, **(b)** cross-sectional SEM and **(c)** TEM images, **(d)** AFM topology images of $NaHCO_3$-modified membranes of the pristine and modified membranes.

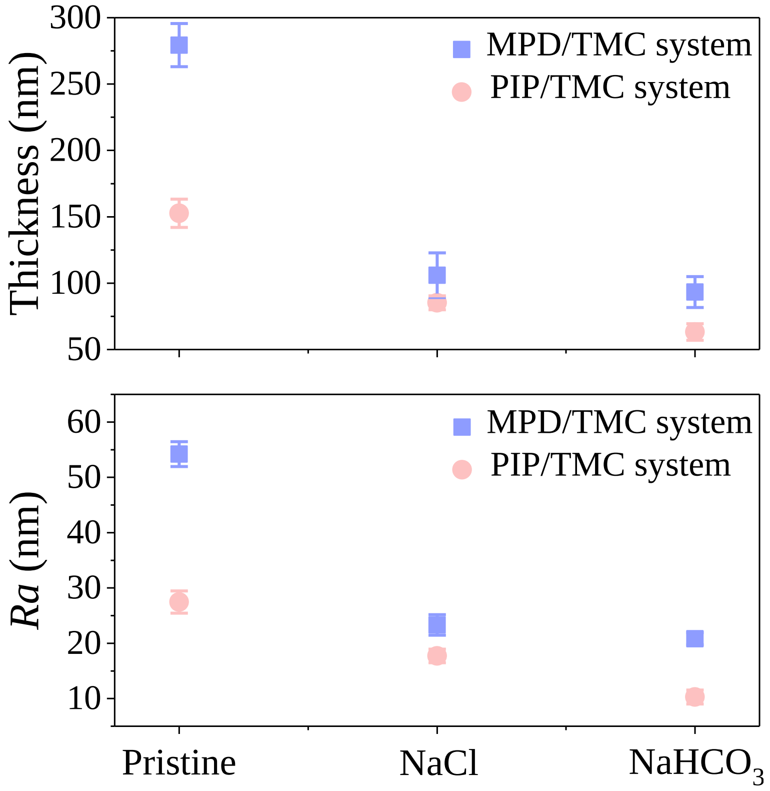

Supplementary Figure 10. PA layer thickness based on TEM results and surface roughness of the pristine and modified membranes. The error bars represent the standard deviation and were calculated on the basis of at least ten data points measured from different samples.PA layer thickness is measured based on TEM images. It can be seen that both the thickness and roughness of salt-modified membranes sharply decrease compared to those of the pristine membrane.

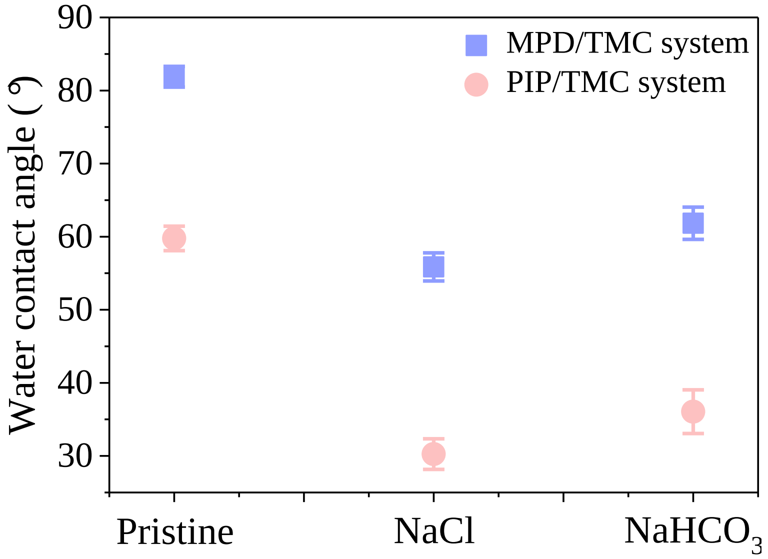

Supplementary Figure 11. Water contact angle of the pristine and modified membranes. The error bars represent the standard deviation and were calculated on the basis of at least ten data points measured from different samples.

a

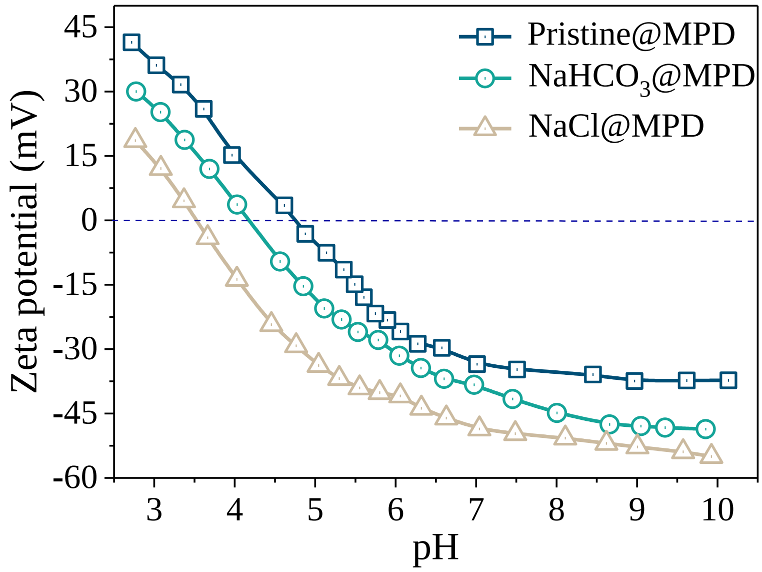

b

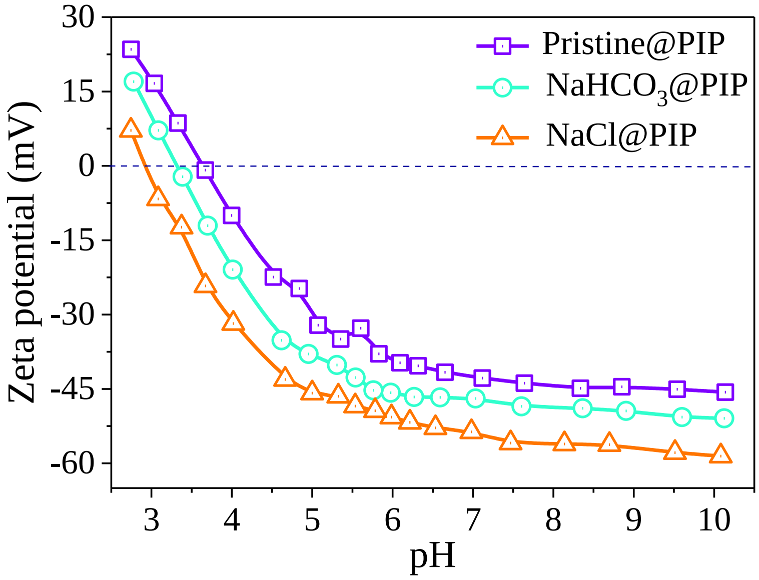

Supplementary Figure 12. Surface zeta potentials of the pristine and modified membranes as a function of pH. **(a)** MPD/TMC system, and **(b)** PIP/TMC system. It can be seen that Zeta potentials of modified membranes in both systems are lower than that of the pristine membrane at the same solution pH, indicating more negative charges on the surface of modified PA layers.

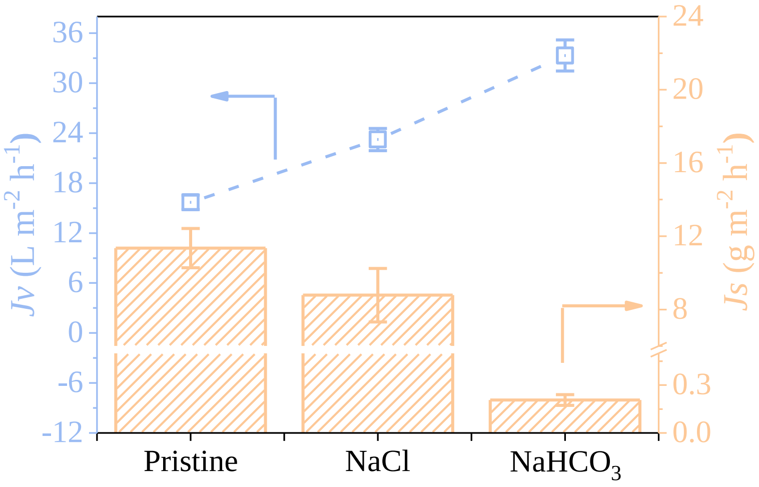

Supplementary Figure 13. FO performance of the pristine and modified membranes under FO mode (PA selective layer facing the feed solution) using DI water and 2 M NaCl as the feed and draw solutions, respectively. The error bars represent the standard deviation and were calculated on the basis of at least three data points measured from different samples. It can be seen that water fluxes follow the order of $NaHCO_3$@MPD > NaCl@MPD > Pristine@MPD, while the reverse salt fluxes present the opposite order, overcoming the permeability-selectivity tradeoff.

a

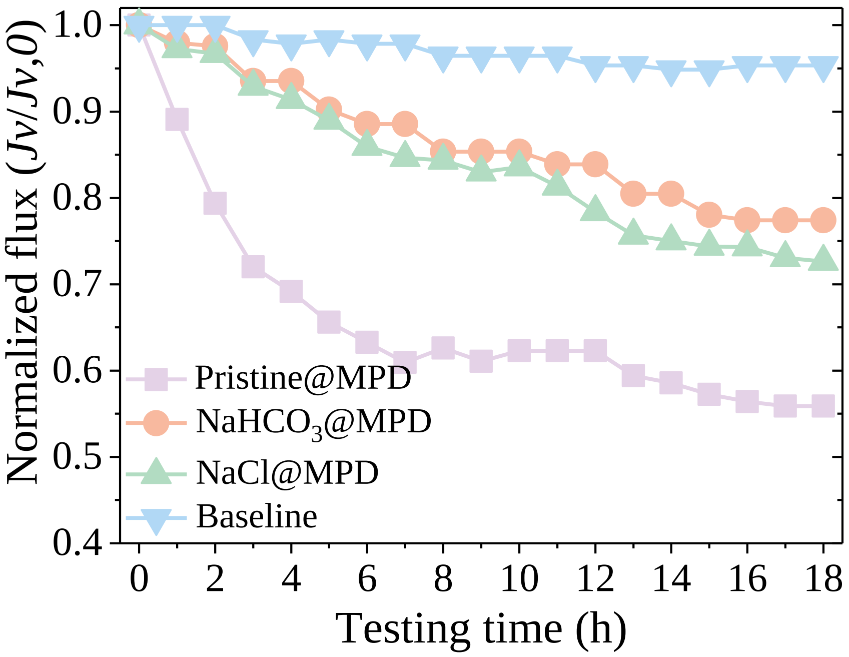

b

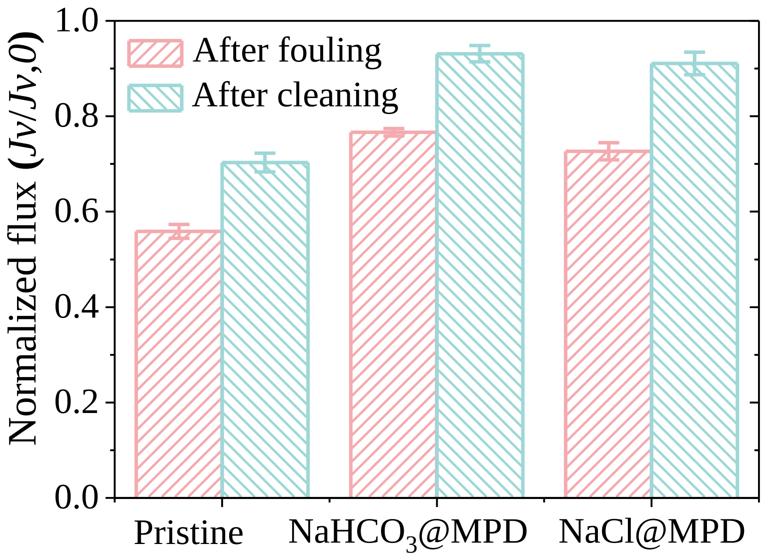

Supplementary Figure 14. Dynamic fouling test results of the pristine and modified membranes under FO model using the synthetic wastewater containing SA and Ca^{2+} ions as the feed solution and 2 M NaCl solution as the draw solution. **(a)** Normalized water flux against the testing time. **(b)** Normalized water flux after fouling and after cleaning. The error bars represent the standard deviation and were calculated on the basis of at least three data points measured from different samples. It can be found that the flux of the pristine membrane drops severely and whose flux

recovery after cleaning is only around 70%. Instead, modified membranes exhibit the steadier and less flux decline as well as the higher flux recovery ratios (~91% to 93%), indicating the improved fouling resistance.

a

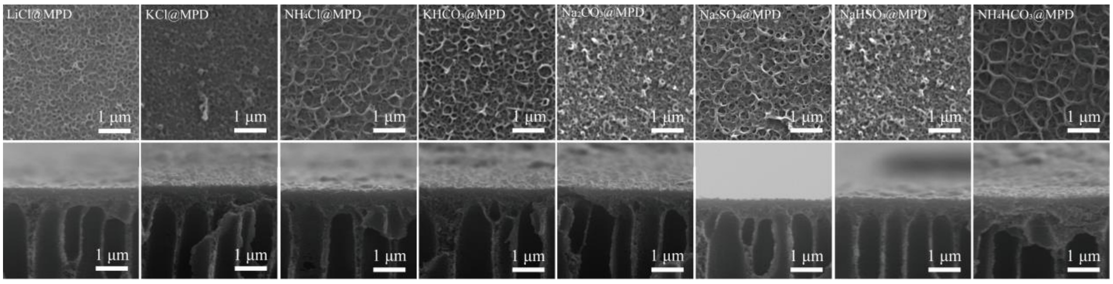

b

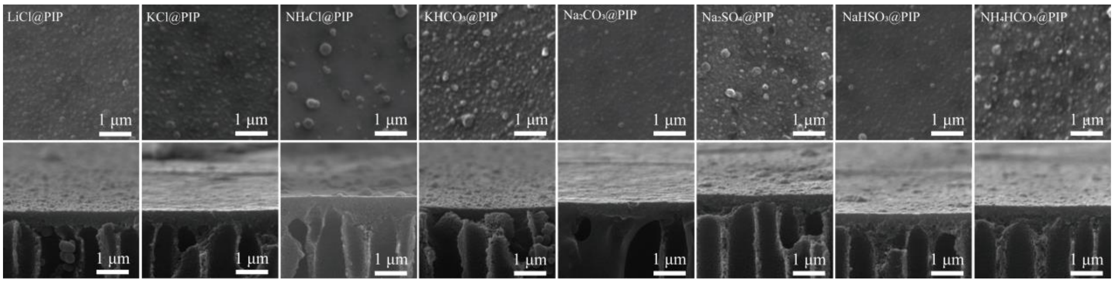

Supplementary Figure 15. SEM images of various salts modified membranes. (a) MPD/TMC monomer system; and **(b)** PIP/TMC monomer system. Supplementary Figure 15a shows that all modified membrane shows the honey-bomb-like Turing surface and thin PA layers. On the other side, Supplementary Figure 15b shows that all modified membranes show the nodular-like surface structures and thin PA layers.

a

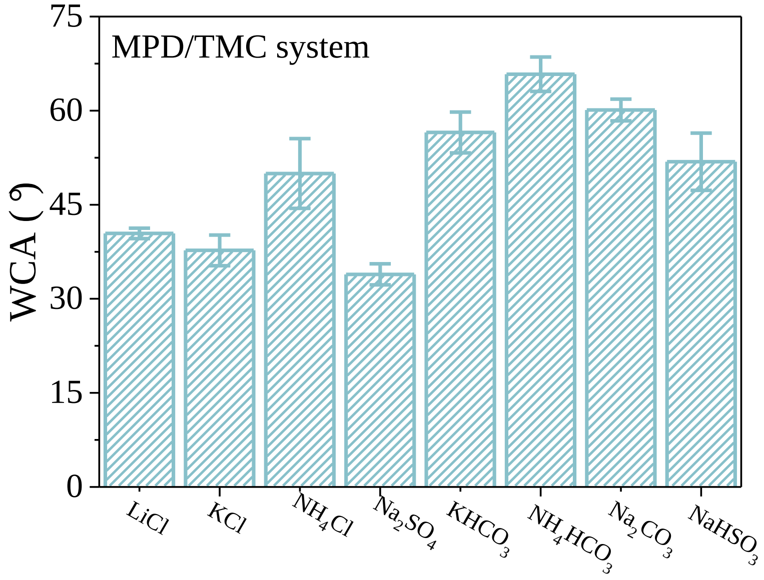
MPD/TMC system
WCA (°)
0
15
30
45
60
75
LiCl
KCl
NH_4Cl
Na_2SO_4
$KHCO_3$
NH_4HCO_3
Na_2CO_3
$NaHSO_3$

b

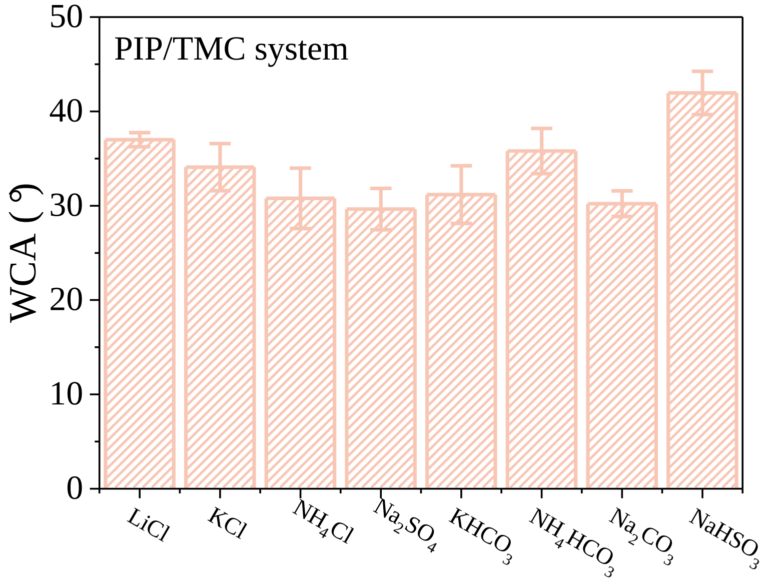
PIP/TMC system
WCA (°)
0
10
20
30
40
50
LiCl
KCl
NH_4Cl
Na_2SO_4
$KHCO_3$
NH_4HCO_3
Na_2CO_3
$NaHSO_3$

c

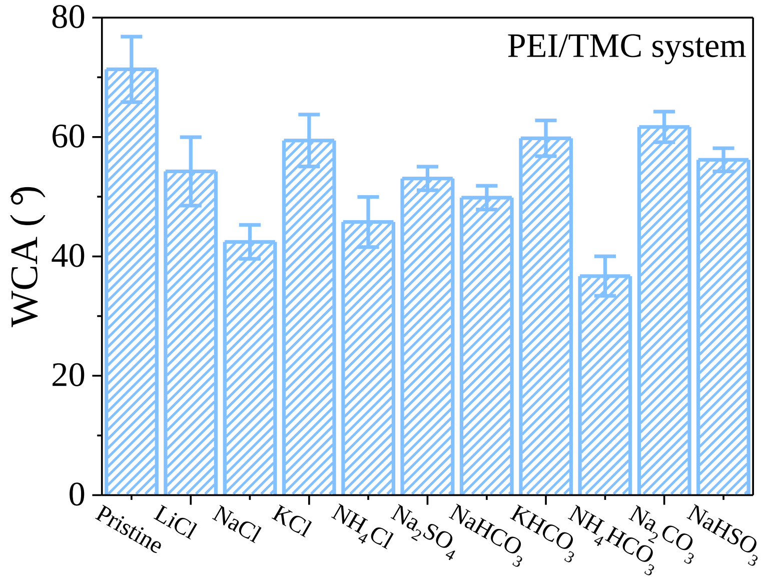

Supplementary Figure 16. Water contact angles (WCA) of various salt-modified membranes. (a) MPD/TMC monomer system, **(b)** PIP/TMC monomer system, and **(c)** PEI/TMC monomer system. The error bars represent the standard deviation and were calculated on the basis of at least ten data points measured from different samples. It can be found that the WCA values of modified membranes are all smaller than that of the pristine membrane.

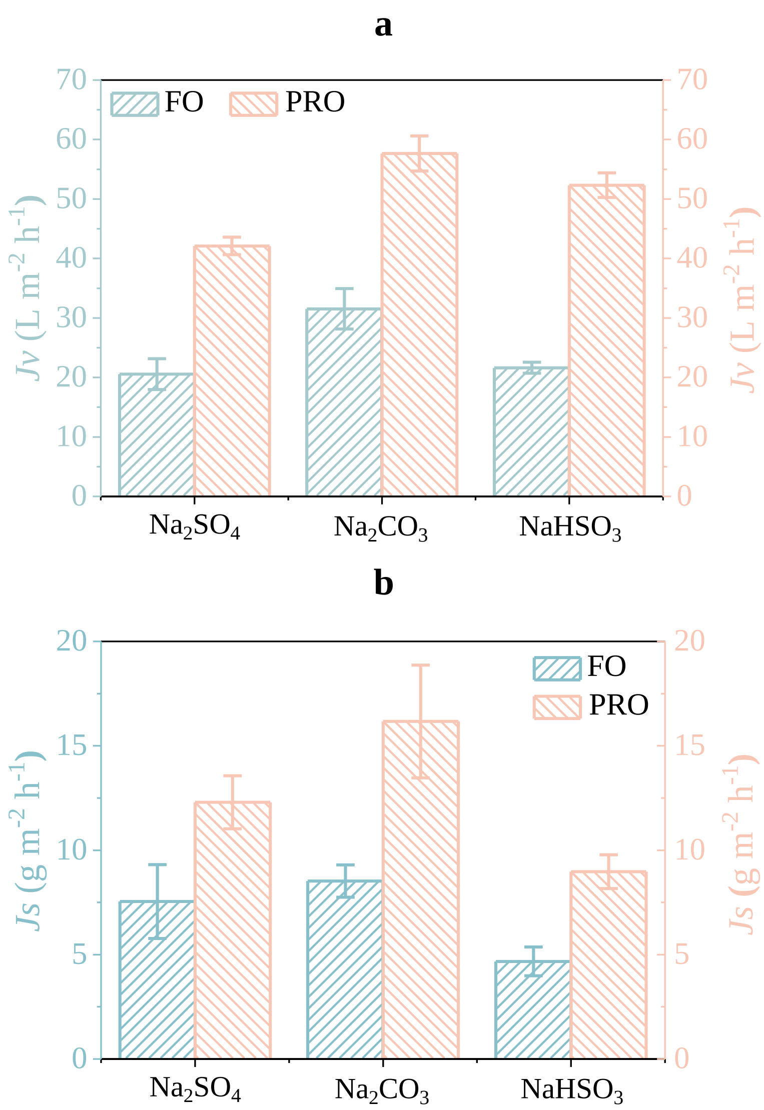

Supplementary Figure 17. FO performance of modified membranes formed by TMC/MPD. (a) Water fluxes and **(b)** reverse salt fluxes of various salt-modified membranes formed by MPD and TMC under FO and PRO modes using DI water and 2 M NaCl as the feed and draw solutions, respectively. The error bars represent the standard deviation and were calculated on the basis of at least three data points measured from different samples. It can be seen that both the water flux and reverse salt flux are influenced by the salt type added in the amine solution.

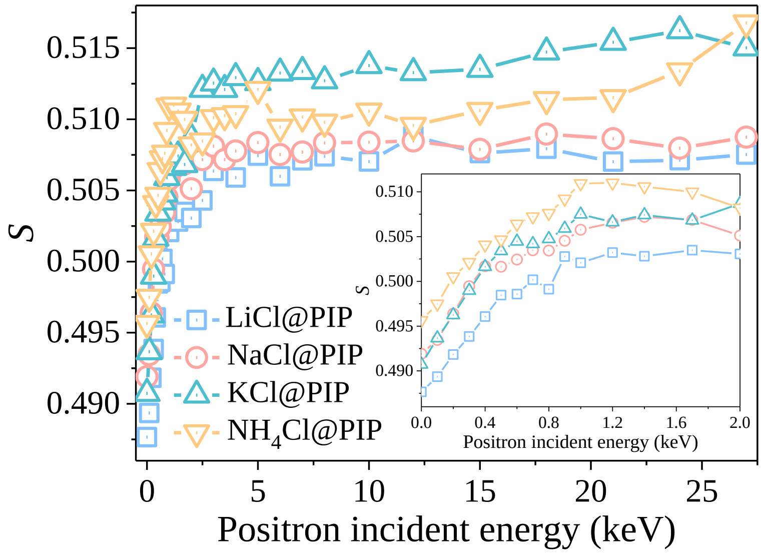

Supplementary Figure 18. *S* profiles of different neutral salts modified membranes formed by PIP and TMC versus the positron incident energy. It can be found that *S* values in range of 0.5 – 1.5 keV follows the order of LiCl@PIP < NaCl@PIP < KCl@PIP < NH_4Cl@PIP.

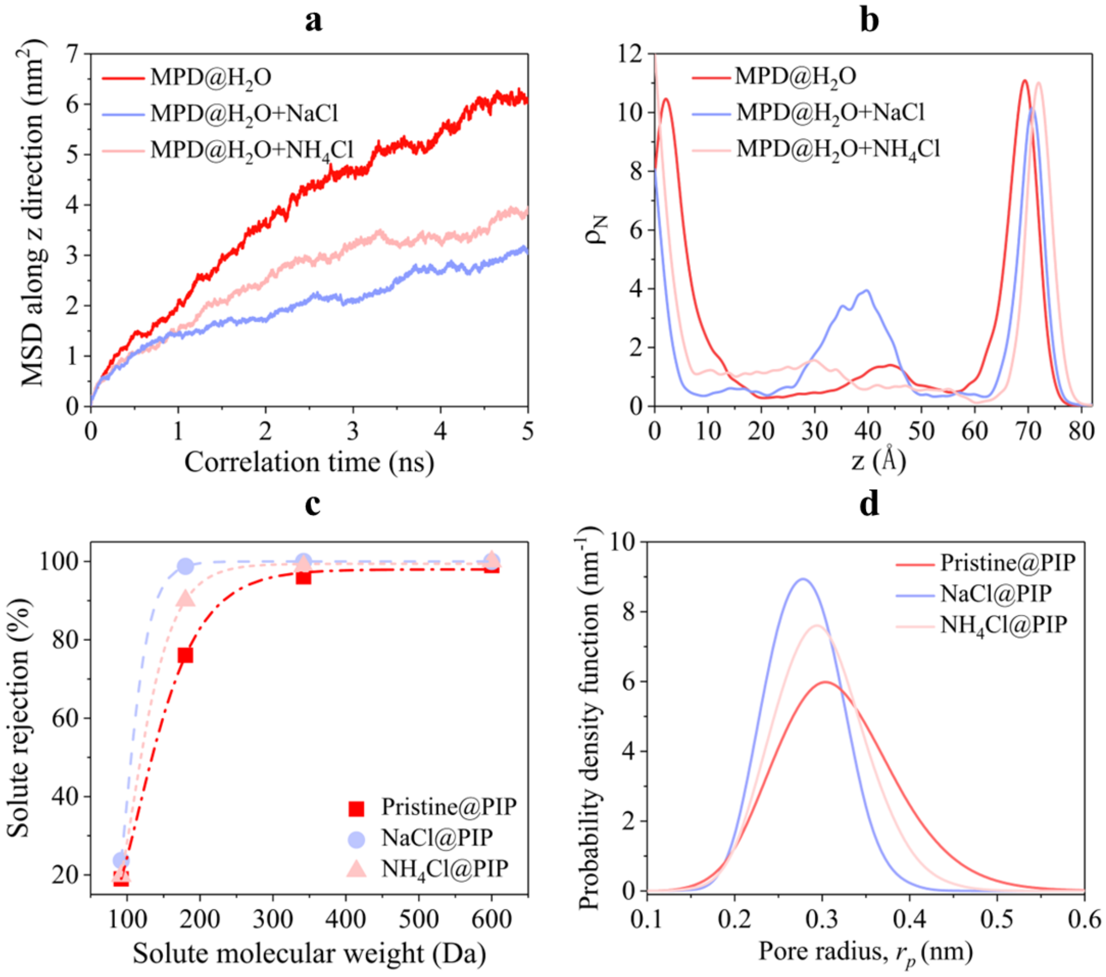

Supplementary Figure 19. MD simulation and neutral solute rejection test results. **(a)** MSD profiles of MPD molecules in systems of pure MPD, MPD+NaCl, and MPD+NH_4Cl. **(b)** The number density (ρN) distribution of MPD molecules in the two systems of pure MPD, MPD+NaCl, and MPD+NH_4Cl. **(c)** Solute rejection as a function of solute molecular weight and **(d)** pore size distribution of membranes Pristine@PIP, NaCl@PIP, NH_4Cl@PIP.

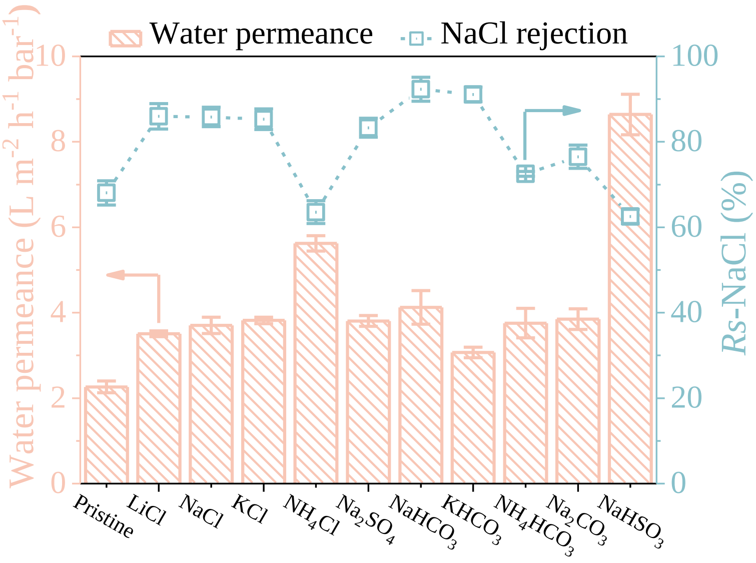

Supplementary Figure 20. Separation performances of the pristine and various salts modified membranes formed by PEI and TMC. The error bars represent the standard deviation and were calculated on the basis of at least three data points measured from different samples. It can be seen that both the water permeance and NaCl rejection are higher than or comparable to those of the pristine membrane.

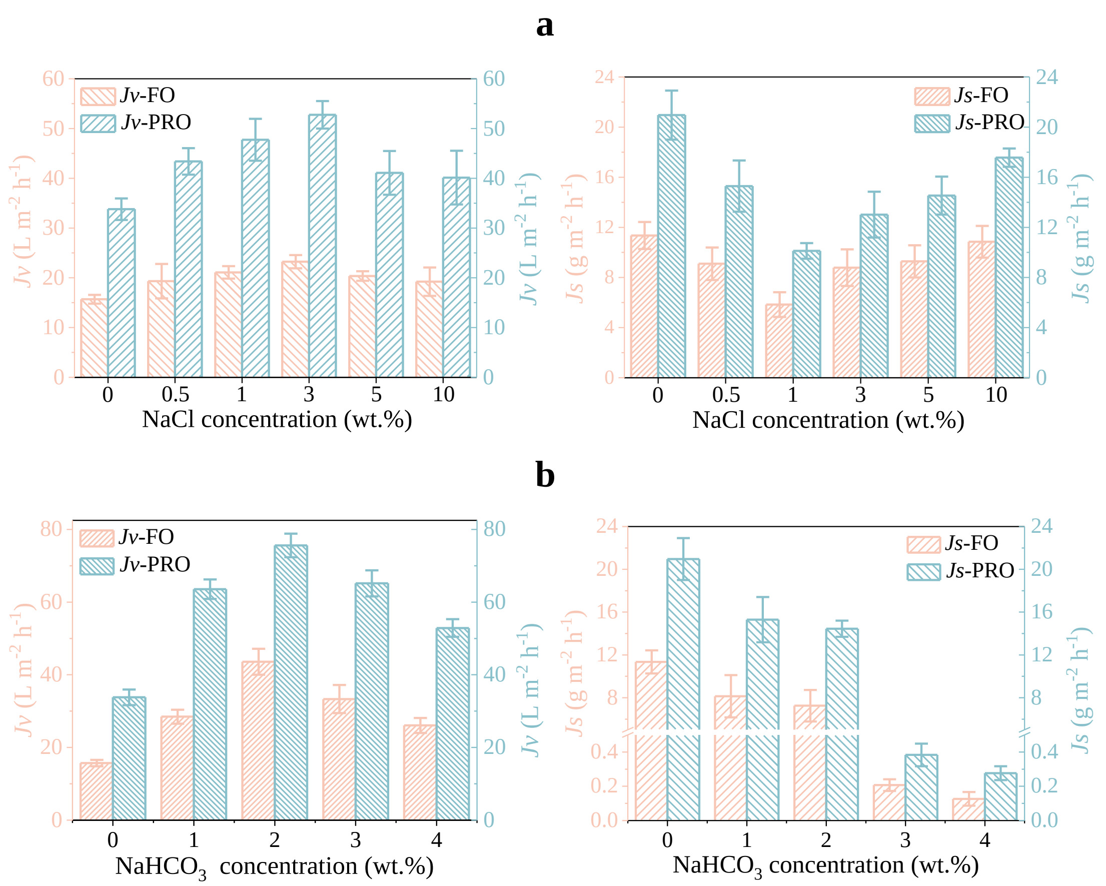

Supplementary Figure 21. FO performances of TFC membranes formed by MPD and TMC modified with various salt concentrations. (a) NaCl and **(b)** $NaHCO_3$-modified membranes using DI water and 2 M NaCl solution as the feed and draw solutions, respectively. The error bars represent the standard deviation and were calculated on the basis of at least three data points measured from different samples. Supplementary Figure 21a shows that the water flux increases initially (till 3 wt.%) and then decreases as the increase of NaCl content, while the reverse salt flux decreases and increases as a function of NaCl content. Supplementary Figure 21b shows that the water flux increases with the increasing $NaHCO_3$ content till 2 wt.% and then decreases with further increment of $NaHCO_3$ content, but the reverse salt flux presents a continuous decline with the increase of $NaHCO_3$ content. Results presented here suggest the optimized salt content is about 3 wt.%. Therefore, the salt content is fixed at 3 wt.% for other salts employed to prepare modified membranes.

3. Supplementary Tables

Supplementary Table 1. Summary of reported salt additives added in aqueous amine solution for fabricating TFC membranes.

Additive category	Additives	Aqueous monomer	Key findings about salt effects	Refs.
Organic salts	TEBAB/TMBAB/ TEBAC	MPD/ PIP	1) Phase transfer catalysts improved the polymerization efficiency in interfacial polymerization by helping the monomer in the water phase move into the organic layer.	[1]
	CSA-TEA	MPD	1) TEA acted as a catalyst to accelerate the MPD–TMC reaction by neutralizing HCl produced during amide formation.<br>2) The addition of CSA protected the microporous skin layer of the support membrane from annealing during curing.	[2]
	TEA+SDS+BMIC/ OMIC	PIP	1) OMIC had a large polar head and a long hydrophobic tail in the structure, playing the role of a surfactant.<br>2) BMIC as a phase transfer catalyst, not only improved the transfer mechanism across the interface, but also increased the diffusion rate.	[3]
	SDS+CTAB	PIP	1) The porosity of thin layers decreased in the presence of CTAB and SDS.<br>2) Surfactant-modified membranes showed a dense and compressed thin layer.	[4]
	TEAC/TBAB/	PIP	1) Amine salt containing larger steric configuration cationic amine group resulted	[5]

	CSA-TEA/BMMIC		in a TFC membrane with better performance.<br>2) The extent of cross-linking was increased with the increasing molecular weight of cationic amine groups.	
	TEAC/TBAB/<br>CSA-TEA/BTMAC/<br>BTEAC/DTMAC	PIP	1) A phase transfer additive should have suitable organic structures and be loose enough to ensure a high capacity to absorb anionic or complex agent and acted as a phase transfer catalyst.<br>2) The ammonium salt with more and longer lipophilic alkyl groups showed the higher catalytic efficiency.<br>3) The larger ammonium salt complex would take over larger space in polymer, and result in a larger free volume.	[6]
	Surfactant + amine salt	Amine	The salt effect was not stated.	[7-9]
	Amine salt	Amine	The salt effect was not stated.	[10]
	Surfactant + amine salt	Amine	The salt effect was not stated.	[11]
	Tertiary amine salt	Amine	1) Tertiary amine acted as both pore forming agent and catalyst (absorbing the IP reaction by-products).	[12-14]
**Inorganic salts**	LiBr + SDS<br>(Alcohol amine)	TEOA/<br>MDEOA	1) The surface roughness and coverage varied with the variation of LiBr concentration.<br>2) Li^{+} ions induce an increased interaction coefficient between TMC and TEOA,	[15]

		forming a dense skin layer.<br>3) Complexation between Li^+ ion and the carbonyl in TMC causes the hydrolysis of acid chloride groups of TMC, inducing a hydrophilic and loose surface layer.	
$CaCl_2$	TEPA	1) The addition of $CaCl_2$ enhanced the interfacial tension and reduced the mass transfer of TEPA to the organic phase.<br>2) Higher $CaCl_2$ content induced an unevenly distributed interfacial polymerization reaction, resulting in the increased surface roughness.	[16]
TEA+NaCl (Nanofiber support)	PIP	1) The addition of salt to the aqueous phase increased the interfacial tension, depressing the mass transfer of PIP and constructing a loose PA layer.<br>2) Further increase NaCl content induced the uneven distribution in nanoscale of the IP reaction, leading to the morphological structure transition from the crisscrossed ridge networks to crowed nodular arrays.	[17]
$NaHCO_3$	MPD	1) Under different pH conditions, different amounts of CO_2 nanobubbles were generated resulting in different morphological features and separation properties.<br>2) Under different concentration conditions, the resulting membranes showed different morphological features, thus tuning the roughness and separation properties.	[18, 19]

NaCl (Heat treatment)	PIP	1) The growth of inorganic salt crystals stretched the nascent and flexible PA layer, and the inorganic salt crystals can sacrifice themselves in water, contributing to the formation of a thin and rough PA layer with the crumpled nanostructure.<br>2) The density and size of NaCl crystals increase with the increasing NaCl concentration, resulting in unidirectional scattering distribution to interconnected dendritic distribution.	[20]

Supplementary Table 2. Surface elemental compositions of the $NaHCO_3$- and NaCl-modified membranes with and without washing by EDX analysis. It can be found that Na and/or Cl content in the $NaHCO_3^-$ and NaCl-modified FO membranes sharply decrease after washing.

Code	C	O	N	Na	Cl
$NaHCO_3$@MPD-unwashed	52.88	27.68	13.78	5.66	/
$NaHCO_3$@MPD-washed	58.07	26.92	13.21	1.80	/
NaCl@MPD-unwashed	61.32	21.35	11.38	3.40	2.54
NaCl@MPD-washed	64.64	23.11	10.92	0.94	0.39

Supplementary Table 3. Na content after 1-day and 6-day washing by AAS detection of TFC membranes and free-standing PA films. It can be found that the salt content trapped in the PA layer after 1-day and 6-day washing decreases slightly from 0.0902/0.0888 to 0.0871/0.0856 μg/cm^2 of membranes NaCl@MPD/$NaHCO_3$@MPD, respectively. On the other side, the content of trapped salt in the free-standing PA layer (NaCl@MPD) reduces slightly from 161.455 mg/kg to 157.280 mg/kg for 1-day and 6-day washing. It can be deduced that a large amount of salt removed on the first day is generally the salt trapped in the substrate membrane as well as between the substrate membrane and the PA layer. However, after the first-day washing, the salt trapped in the PA layer can be partially washed out with a longer washing duration (6 days) due to the complexion between the salt ions and the carbonyl groups of the PA network[15-17,23,24]. The ultimate salt content trapped accounts for 0.016% of the weight of the PA layer.

Membrane code	Na content (μg cm^{-2})	Film code	Na content (mg kg^{-1})
NaCl@MPD-1 day	0.0902	NaCl@MPD-1 day	161.455
NaCl@MPD-6 day	0.0871	NaCl@MPD-6 day	157.280
$NaHCO_3$@MPD-1 day	0.0888	/	/
$NaHCO_3$@MPD-6 day	0.0856	/	/

Supplementary Table 4. Surface elemental compositions of pristine and modified membranes by XPS analysis. Commonly, the detection depth of XPS technique for polymeric material is less than 10 nm[21], hence the detected O/N ratio actually only refers to the elemental composition of the top skin of the PA layer, which should be the region formed in the diffusion-limited growth regime (relatively loose part). To detect the middle part of the resulting PA layers, the in-depth profile of elemental compositions was therefore further measured by XPS using $C_{24}H_{12}^{+}$ ion source to etch membranes. The results in Supplementary Table 4 reveal that the modified membrane possesses a relatively looser top skin (high O/N ratio) compared to that of the pristine membrane. With a deeper detection depth, the position might reach the middle part of the PA layer in the modified membrane, exhibiting lower O/N ratios (higher crosslinking degree). In comparison with the pristine membrane, the O/N ratio of the modified membrane is even slightly lower than that of the pristine membrane, suggesting the formation of a relatively denser middle layer. Meanwhile, Na content in the modified membrane decreases with the deeper detection depth.

Membrane code	C	O	N	Na	Cl	O/N
Pristine@MPD	71.84	16.83	11.32	/	/	1.49
$NaHCO_3$@MPD	69.25	18.51	11.26	0.98	/	1.64
NaCl @MPD	71.15	15.79	9.93	1.66	1.47	1.59
Pristine@PIP	72.92	15.36	11.72	/	/	1.31
$NaHCO_3$@PIP	71.3	16.74	10.82	1.14	/	1.55
NaCl@PIP	70.42	17.17	11.28	0.37	0.76	1.52
Pristine@MPD-0nm	72.53	16.59	10.88	/	/	1.53
Pristine@MPD-20nm	72.31	16.16	11.53	/	/	1.40

Pristine@MPD-40nm	74.03	14.95	11.02	/	/	1.36
Pristine@MPD-60nm	73.12	15.65	11.23	/	/	1.39
Pristine@MPD-80nm	73.39	15.03	11.58	/	/	1.30
NaCl@MPD-0nm	70.71	15.93	10.25	1.38	1.73	1.55
NaCl@MPD-20nm	72.40	14.56	11.13	0.73	1.18	1.31
NaCl@MPD-40nm	72.58	14.62	11.46	0.47	0.87	1.28
NaCl@MPD-60nm	72.32	14.89	11.77	0.40	0.62	1.27
NaCl@MPD-80nm	72.69	14.85	11.58	0.29	0.59	1.28

Supplementary Table 5. Free volume pore properties of the pristine and modified membranes (error bars represent the standard deviation and were calculated on the basis of at least three data points measured from different samples). It summarizes the free volume pore properties of the pristine and modified membranes formed by MPD/TMC or PIP/TMC, including *o*-Ps lifetime (τ_3), free volume pore intensity (I_3), free volume pore size (R), and fraction free volume (FFV). It can be found that τ_3 and R values of modified membranes are all smaller than that of the pristine membrane, indicating the smaller free volume pore size in the modified PA layers. In contrast, I_3 values of modified membranes are all larger than that of the pristine membrane, demonstrating more free volume pores in the modified PA layers. Accordingly, modified membranes show the smaller FFV values, suggesting the denser structures of modified PA layers.

Membrane Code	τ_3 (ns)	I_3 (%)	R (Å)	FFV (%)
Pristine@MPD	1.616±0.036	13.697±0.563	2.473±0.035	1.562±0.131
NaCl@MPD	1.422±0.049	15.353±0.497	2.311±0.054	1.428±0146
$NaHCO_3$@MPD	1.396±0.042	16.874±0.462	2.183±0.050	1.323±0.127
Pristine@PIP	1.922±0.051	12.971±0.368	2.783±0.041	2.107±0.153
NaCl@PIP	1.537±0.038	16.108±0.417	2.386±0.040	1.649±0.125
$NaHCO_3$@PIP	1.483±0.047	14.448±0.483	2.324±0.051	1.367±0.136

Supplementary Table 6. Polymer density of free-standing films of Pristine@MPD and NaCl@MPD measured by a pycnometer (error bars represent the standard deviation and were calculated on the basis of at least three data points measured from different samples).

Film code	Density (g cm^{-3})
Pristine@MPD	1.183±0.017
NaCl@MPD	1.238±0.023

Supplementary Table 7. Properties of different ions. Supplementary Table 7 summarizes intrinsic properties, such as diffusion coefficient, ionic radius and hydrated radius, of different ions employed in this work, including Li^+, Na^+, K^+, NH_4^+, Cl^- and SO_4^{2-}. It can be found that the water permeance of the modified membrane is positively correlated to the ionic radius of the salt rather than the hydrated radius of the cationic ion.

Ion Type	Diffusion coefficient (10^{-9} m s^{-1})	Ionic radius (nm)	Hydrated radius (nm)
Li^+	1.029	0.060	0.382
Na^+	1.334	0.102	0.358
K^+	1.957	0.138	0.275
NH_4^+	1.760	0.161	0.331
Cl^-	2.030	0.181	0.332
SO_4^{2-}	1.060	0.230	0.379

Supplementary Table 8. FO performance comparison with reported lab-made and commercial FO membranes. 2 M NaCl solution and DI water were used as the draw and feed solutions under PRO mode.

Membrane code	*Jv* (L m^{-2} h^{-1})	*Js* (g m^{-2} h^{-1})	Ref.
$NaHCO_3$@MPD	62.8	0.4	This work
PA-2-HMTA	50.6	7.5	[22]
PA-AMPES/3	56.3	9.5	[23]
PA-PES/NMP	62.7	10.3	[24]
PA-PES/SPES	42.1	11.1	[25]
PA-PSF/HNT	54.0	31.0	[26]
PA-PEI-DTPMP	62.0	23.0	[27]
PA-SPPSU	48.0	7.6	[28]
M-T/CNT-PA	45.0	7.0	[29]
TFC-D3	60.5	16.5	[30]
PA-1-TAEA-9	57.9	22.4	[31]
PESU-co-sPPSU	33.0	2.8	[32]
TFN0.1	36.5	1.5	[33]
TFN0.6	59.4	31.0	[34]
HTI-TFC	24.0	10.8	[35]
HTI-CTA	18.5	20.0	

Supplementary Table 9. Intrinsic separation performance comparison with reported lab-made and commercial FO membranes.

Membrane code	Water permeance (L m^{-2} h^{-1} bar^{-1})	NaCl rejection (%)	Testing condition	Ref.
$NaHCO_3$@MPD	2.9	99.2	1000 ppm, 5 bar	This work
M-T/CNT-PA	2.0	98.0	200 ppm, 5 bar	[29]
TFC-G_{10}	2.1	94.3	1000 ppm, 5 bar	[36]
MT-5	2.3	92.9	10 mM, 2 bar	[37]
TFN-0.25GO	1.9	98.7	1000 ppm, 10 bar	[38]
TFC-D3	1.6	97.7	2000 ppm, 8.6 bar	[30]
TFN0.5	2.0	92.7	20 mM, 2.5 bar	[39]
TFC-sPPSU	2.0	91.0	1000 ppm, 1 bar	[40]
TFC-D4	1.8	97.9	2000 ppm, 8.6 bar	[41]
TFC-PES	1.8	81.5	200 ppm, 1 bar	[24]
Double-skin TFC	1.3	88.3	2000 ppm, 5 bar	[42]
TFC PDA@PSf-5h	0.5	88.0	200 ppm, 2 bar	[43]
AMTFC-3	1.1	96.6	1000 ppm, 6 bar	[23]
MT-75	1.2	93.6	10 mM, 3 bar	[44]
GOT-0.25	1.8	98.7	1000 ppm, 10 bar	[38]
TFC-sPPSU	0.7	91.5	400 ppm, 3.45 bar	[32]
TFC2	1.8	93.4	20 mM, 3.75 bar	[45]
CTA-HW	1.2	78.5		
CTA-W	0.3	81.9		
CTA-NW	0.5	92.4		
HTI	0.7	89.1	2000 ppm, 8.6 bar	[41]
Aquaporin	0.5	97.9	2000 ppm, 125 psi	[46]

Supplementary Table 10. NF performance comparison with reported lab-made and commercial NF membranes.

Membrane code	Water permeance (L m^{-2} h^{-1} bar^{-1})	NaCl Rejection (%)	Testing condition	Ref.
NaCl@PIP	16.6	72.7	5 bar, 1000 ppm	This work
$NaHCO_3$@PIP	12.4	75.0		
Na_2SO_4@PIP	15.7	60.3		
TFC-24	2.1	47.8	10.3 bar, 1000 ppm	[47]
TFC-15	5.8	35.5		
TFC-12	8.3	44.6		
M4	4.9	70.0	10 bar, 1000 ppm	[48]
M9	8.3	50.0		
CSA	4.8	30.4	10 bar, 2000 ppm	[49]
CSA/GO	5.5	33.0		
CSA/TiO_2	5.1	35.6		
CSA/rGo-TiO_2	6.1	36.6		
GO-COCl	3.8	57.2	6 bar, 1000 ppm	[50]
MWCNT-COOH	6.2	34.0	6 bar, 2000 ppm	[51]
MWCNT-OH	6.9	35.3		
MWCNT-NH	5.3	35.1		
PMMA-MWCNT	7.0	44.1	10 bar, 2000 ppm	[52]
TS-I	13.3	51.2	4.8 bar, 2000 ppm	[53]
PIP	12.6	15.0	4 bar, 1000 ppm	[54]
SDS	17.1	27.0		
SDBS	14.9	21.3		
SB3-14	20.3	20.9		
CTAB	26.2	9.9		
TsNA	14.9	14.8		
PIP0.05-SLS	23.1	45.0	5 bar, 2000 ppm	[55]
PIP0.1-SLS	16.4	42.1		

TFC-2	21.0	18.8	5 bar, 2000 ppm	[56]
NF-0.1%CB-1	15.5	12.5	6 bar, 1000 ppm	[57]
EIP	16.6	30.0	5 bar, 1000 ppm	[58]
TFN0.3	2.4	59.5	8 bar, 1000 ppm	[59]
TFC-PDA/PEI	10.5	50.0	6 bar, 1000 ppm	[60]
PA/PES/PANI-0.2	15.7	33.0	6 bar, 2000 ppm	[61]
CNT0.005	5.3	36.7	10 bar, 2000 ppm	[62]
NFM-3	5.6	30.0	4 bar, 1000 ppm	[63]
TFNMA-GO-ODA	8.3	38.0	6 bar, 2000 ppm	[64]
NFM-4	9.8	23.5	5 bar, 500 ppm	[65]
TFC-ZIF-8-PSS	9.6	38.0	5 bar, 500 ppm	[66]
PA/PES	12.3	48.0	10 bar, 2000 ppm	[67]
PA-PD/PES	11.0	31.0	2 bar, 1000 ppm	[68]
PA-SiO_2/PES	9.5	26.0	6 bar, 2000 ppm	[69]
NF270	11.0	66.0	5 bar, 2000 ppm	
NTR-7450	11.0	50.0	10 bar, 2000 ppm	
NTR-7410	18.0	10.0	10 bar, 2000 ppm	[70]
NTR-7470	4.0	70.0	10 bar, 2000 ppm	
NTR-7250	4.0	50.0	10 bar, 2000 ppm	
XN-45	5.0	25.0	7.6 bar, 2000 ppm	
Koch TFC-SR2	7.5	24.0	10 bar, 585 ppm	[71]
Koch TFC-SR3	2.2	38.0	10 bar, 585 ppm	
GE-Osmonics HL	6.9	33.0	7.6 bar, 1000 ppm	[72]

Supplementary Table 11. NF performance comparison with reported lab-made and commercial NF membranes.

Membrane code	Water permeance (L m^{-2} h^{-1} bar^{-1})	Na_2SO_4 Rejection (%)	Testing condition	Ref.
LiCl@PIP	12.6	99.2	5 bar, 1000 ppm	This work
NaCl@PIP	16.6	97.8		
KCl@PIP	17.5	97.4		
$NaHCO_3$@PIP	12.4	99.2		
GO-COCl	3.8	97.1	6 bar, 1000 ppm	[50]
MWCNT-COOH	6.2	96.6	6 bar, 2000 ppm	[51]
MWCNT-OH	6.9	97.6		
MWCNT-NH	5.3	96.8		
PMMA-MWCNT	7.0	99.0		[52]
TS-I	12.7	99.1	4.8 bar, 2000 ppm	[53]
PIP	12.6	97.1	4 bar, 1000 ppm	[54]
SDBS	14.9	98.8		
TsNA	14.9	98.0		
NF-0.1%CB-1	15.5	98.0	6 bar, 1000 ppm	[57]
EIP	16.6	95.5	5 bar, 1000 ppm	[58]
TFC-PDA/PEI	10.5	97.0	6 bar, 1000 ppm	[60]
CNT0.005	5.3	95.7	10 bar, 2000 ppm	[62]
NFM-3	5.6	98.8	4 bar, 1000 ppm	[63]
TFNMA-GO-ODA	8.3	98.4	6 bar, 2000 ppm	[64]
NFM-4	9.8	95.0	5 bar, 500 ppm	[65]
TFC-ZIF-8-PSS	9.6	91.0	5 bar, 500 ppm	[66]
PA-PD/PES	11.4	93.5	2 bar, 1000 ppm	[68]
PA-SiO2/PES	9.5	97.4	6 bar, 2000 ppm	[69]
PA-TEOS0.5	15.9	92.0	5 bar, 500 ppm	[73]
PA-Flower	6.4	89.9	10 bar, 1000 ppm	[74]
TFN/UiO-66-NH2	13.0	98.1	6 bar, 1000 ppm	[75]

TFC NFM	17.6	95.0	6 bar, 1000 ppm	[76]
NF90	7.0	99.1	3.5 bar, 1000 ppm	[77]
NF270	11.2	98.5		

Supplementary Table 12. Detailed fabrication parameters of the TFC membranes

System		MPD/TMC	PIP/TMC	PEI/TMC
**Substrate membrane**	Dope composition (wt.%)	PSF/PEG-400/NMP =18/16/66		
**Active layer**	Aqueous phase composition (wt.%)	MPD/H_2O 3.4/96.6 MPD/salt/H_2O 3.4/3/93.6	PIP/H_2O 0.35/99.65 MPD/salt/H_2O 0.35/3/96.65	PEI/H_2O 2/98 MPD/salt/H_2O 2/3/95
	Organic phase composition (w/v%)	TMC/Hexane 0.15/100	TMC/Hexane 0.15/100	TMC/Hexane 0.15/100
	Immersion time in amine solution (min)	5	10	30
	IP reaction time (min)	1	2	10

4. Supplementary References

[1] J. Jegal, S.G. Min, K.H. Lee, Factors affecting the interfacial polymerization of polyamide active layers for the formation of polyamide composite membranes, Journal of Applied Polymer Science, 86 (2002) 2781-2787.

[2] A.K. Ghosh, B.-H. Jeong, X. Huang, E.M. Hoek, Impacts of reaction and curing conditions on polyamide composite reverse osmosis membrane properties, Journal of Membrane Science, 311 (2008) 34-45.

[3] L. Yung, H. Ma, X. Wang, K. Yoon, R. Wang, B.S. Hsiao, B. Chu, Fabrication of thin-film nanofibrous composite membranes by interfacial polymerization using ionic liquids as additives, Journal of Membrane Science, 365 (2010) 52-58.

[4] Y. Mansourpanah, K. Alizadeh, S. Madaeni, A. Rahimpour, H.S. Afarani, Using different surfactants for changing the properties of poly (piperazineamide) TFC nanofiltration membranes, Desalination, 271 (2011) 169-177.

[5] J. Xiang, Z. Xie, M. Hoang, K. Zhang, Effect of amine salt surfactants on the performance of thin film composite poly (piperazine-amide) nanofiltration membranes, Desalination, 315 (2013) 156-163.

[6] J. Xiang, Z. Xie, M. Hoang, D. Ng, K. Zhang, Effect of ammonium salts on the properties of poly (piperazineamide) thin film composite nanofiltration membrane, Journal of membrane science, 465 (2014) 34-40.

[7] J.E. Tomaschke, I.E. Ary, Interfacially synthesized reverse osmosis membranes and processes for preparing the same, in, Google Patents, 1993.

[8] J.E. Tomaschke, Interfacially synthesized reverse osmosis membranes and processes for preparing the same, in, Google Patents, 1993.

[9] J.E. Tomaschke, Interfacially synthesized reverse osmosis membrane containing an amine salt and processes for preparing the same, in, Google Patents, 1989.

[10] J.E. Tomaschke, Interfacially polymerized, bipiperidine-polyamide membranes for reverse osmosis and/or nanofiltration and process for making the same, in, Google Patents, 2002.

[11] M. Hirose, Composite reverse osmosis membrane and method for producing the same, in, Google Patents, 2004.

[12] J.-y. Koo, J.-E. Kim, W.-J. Kim, K.S. Park, Composite polyamide reverse osmosis membrane and method of producing the same, in, Google Patents, 2001.

[13] J.-y. Koo, Y.S. Yoon, N. Kim, J.-E. Kim, Composite polyamide reverse osmosis membrane and method of producing the same, in, Google Patents, 2002.

[14] J.-y. Koo, Y.S. Yoon, Composite polyamide reverse osmosis membrane and method of producing the same, in, Google Patents, 2000.

[15] B. Tang, C. Zou, P. Wu, Study on a novel polyester composite nanofiltration membrane by interfacial polymerization. II. The role of lithium bromide in the performance and formation of composite membrane, Journal of Membrane Science, 365 (2010) 276-285.

[16] X. Fan, Y. Dong, Y. Su, X. Zhao, Y. Li, J. Liu, Z. Jiang, Improved performance of composite nanofiltration membranes by adding calcium chloride in aqueous phase during interfacial polymerization process, Journal of membrane science, 452 (2014) 90-96.

[17] K. Shen, P. Li, T. Zhang, X. Wang, Salt-tuned fabrication of novel polyamide composite nanofiltration membranes with three-dimensional turing structures for effective desalination, Journal of Membrane Science, 607 (2020) 118153.

[18] L.E. Peng, Z. Yao, X. Liu, B. Deng, H. Guo, C.Y. Tang, Tailoring Polyamide Rejection Layer with Aqueous Carbonate Chemistry for Enhanced Membrane

Separation: Mechanistic Insights, Chemistry-Structure-Property Relationship, and Environmental Implications, Environ Sci Technol, 53 (2019) 9764-9770.

[19] X. Ma, Z. Yang, Z. Yao, H. Guo, Z. Xu, C.Y. Tang, Tuning roughness features of thin film composite polyamide membranes for simultaneously enhanced permeability, selectivity and anti-fouling performance, Journal of colloid and interface science, 540 (2019) 382-388.

[20] Z. Liu, T. Wang, D. Wang, Z. Mi, Regulating the morphology of nanofiltration membrane by thermally induced inorganic salt crystals for efficient water purification, Journal of Membrane Science, 617 (2021) 118645.

[21] J. DeVries, Surface characterization methods—XPS, TOF-SIMS, and SAM a complimentary ensemble of tools, Journal of materials engineering and performance, 7 (1998) 303-311.

[22] L. Shen, L. Tian, J. Zuo, X. Zhang, S. Sun, Y. Wang, Developing high-performance thin-film composite forward osmosis membranes by various tertiary amine catalysts for desalination, Advanced Composites and Hybrid Materials, 2 (2019) 51-69.

[23] M. Qiu, J. Wang, C. He, A stable and hydrophilic substrate for thin-film composite forward osmosis membrane revealed by in-situ cross-linked polymerization, Desalination, 433 (2018) 1-9.

[24] P. Sukitpaneenit, T.-S. Chung, High Performance Thin-Film Composite Forward Osmosis Hollow Fiber Membranes with Macrovoid-Free and Highly Porous Structure for Sustainable Water Production, Environmental Science & Technology, 46 (2012) 7358-7365.

[25] S. Sahebi, S. Phuntsho, Y.C. Woo, M.J. Park, L.D. Tijing, S. Hong, H.K. Shon, Effect of sulphonated polyethersulfone substrate for thin film composite

forward osmosis membrane, Desalination, 389 (2016) 129-136.

[26] M. Ghanbari, D. Emadzadeh, W. Lau, H. Riazi, D. Almasi, A. Ismail, Minimizing structural parameter of thin film composite forward osmosis membranes using polysulfone/halloysite nanotubes as membrane substrates, Desalination, 377 (2016) 152-162.

[27] L. Shen, F. Wang, L. Tian, X. Zhang, C. Ding, Y. Wang, High-performance thin-film composite membranes with surface functionalization by organic phosphonic acids, Journal of membrane science, 563 (2018) 284-297.

[28] N. Widjojo, T.-S. Chung, M. Weber, C. Maletzko, V. Warzelhan, A sulfonated polyphenylenesulfone (sPPSU) as the supporting substrate in thin film composite (TFC) membranes with enhanced performance for forward osmosis (FO), Chemical Engineering Journal, 220 (2013) 15-23.

[29] M. Zhang, W. Jin, F. Yang, M. Duke, Y. Dong, C.Y. Tang, Engineering a nanocomposite interlayer for a novel ceramic-based forward osmosis membrane with enhanced performance, Environmental science & technology, 54 (2020) 7715-7724.

[30] X. Liu, H.Y. Ng, Fabrication of layered silica–polysulfone mixed matrix substrate membrane for enhancing performance of thin-film composite forward osmosis membrane, Journal of Membrane Science, 481 (2015) 148-163.

[31] L. Shen, J. Zuo, Y. Wang, Tris (2-aminoethyl) amine in-situ modified thin-film composite membranes for forward osmosis applications, Journal of Membrane Science, 537 (2017) 186-201.

[32] N. Widjojo, T.-S. Chung, M. Weber, C. Maletzko, V. Warzelhan, The role of sulphonated polymer and macrovoid-free structure in the support layer for thin-film composite (TFC) forward osmosis (FO) membranes, Journal of

Membrane Science, 383 (2011) 214-223.

[33] N. Niksefat, M. Jahanshahi, A. Rahimpour, The effect of SiO2 nanoparticles on morphology and performance of thin film composite membranes for forward osmosis application, Desalination, 343 (2014) 140-146.

[34] D. Emadzadeh, W.J. Lau, T. Matsuura, A.F. Ismail, M. Rahbari-Sisakht, Synthesis and characterization of thin film nanocomposite forward osmosis membrane with hydrophilic nanocomposite support to reduce internal concentration polarization, Journal of Membrane Science, 449 (2014) 74-85.

[35] Q. Long, G. Qi, Y. Wang, Evaluation of renewable gluconate salts as draw solutes in forward osmosis process, ACS Sustainable Chemistry & Engineering, 4 (2016) 85-93.

[36] H. Salehi, A. Shakeri, H. Mahdavi, R.G. Lammertink, Improved performance of thin-film composite forward osmosis membrane with click modified polysulfone substrate, Desalination, 496 (2020) 114731.

[37] X. Zhang, L. Shen, W.-Z. Lang, Y. Wang, Improved performance of thin-film composite membrane with PVDF/PFSA substrate for forward osmosis process, Journal of membrane science, 535 (2017) 188-199.

[38] M.J. Park, S. Phuntsho, T. He, G.M. Nisola, L.D. Tijing, X.-M. Li, G. Chen, W.-J. Chung, H.K. Shon, Graphene oxide incorporated polysulfone substrate for the fabrication of flat-sheet thin-film composite forward osmosis membranes, Journal of Membrane Science, 493 (2015) 496-507.

[39] D. Emadzadeh, W.J. Lau, T. Matsuura, M. Rahbari-Sisakht, A.F. Ismail, A novel thin film composite forward osmosis membrane prepared from PSf–TiO2 nanocomposite substrate for water desalination, Chemical Engineering Journal, 237 (2014) 70-80.

[40] P. Zhong, X. Fu, T.-S. Chung, M. Weber, C. Maletzko, Development of thin-film composite forward osmosis hollow fiber membranes using direct sulfonated polyphenylenesulfone (sPPSU) as membrane substrates, Environmental science & technology, 47 (2013) 7430-7436.

[41] X. Liu, H.Y. Ng, Double-blade casting technique for optimizing substrate membrane in thin-film composite forward osmosis membrane fabrication, Journal of Membrane Science, 469 (2014) 112-126.

[42] P.H. Duong, T.-S. Chung, S. Wei, L. Irish, Highly permeable double-skinned forward osmosis membranes for anti-fouling in the emulsified oil–water separation process, Environmental science & technology, 48 (2014) 4537-4545.

[43] G. Han, S. Zhang, X. Li, N. Widjojo, T.-S. Chung, Thin film composite forward osmosis membranes based on polydopamine modified polysulfone substrates with enhancements in both water flux and salt rejection, Chemical Engineering Science, 80 (2012) 219-231.

[44] X. Zhang, L. Shen, C.-Y. Guan, C.-X. Liu, W.-Z. Lang, Y. Wang, Construction of SiO2@ MWNTs incorporated PVDF substrate for reducing internal concentration polarization in forward osmosis, Journal of Membrane Science, 564 (2018) 328-341.

[45] J. Wei, C. Qiu, C.Y. Tang, R. Wang, A.G. Fane, Synthesis and characterization of flat-sheet thin film composite forward osmosis membranes, Journal of Membrane Science, 372 (2011) 292-302.

[46] L. Xia, M.F. Andersen, C. Hélix-Nielsen, J.R. McCutcheon, Novel commercial aquaporin flat-sheet membrane for forward osmosis, Industrial & Engineering Chemistry Research, 56 (2017) 11919-11925.

[47] P. Veerababu, B.B. Vyas, P.S. Singh, P. Ray, Limiting thickness of polyamide–

polysulfone thin-film-composite nanofiltration membrane, Desalination, 346 (2014) 19-29.

[48] Y. Mansourpanah, S. Madaeni, A. Rahimpour, Fabrication and development of interfacial polymerized thin-film composite nanofiltration membrane using different surfactants in organic phase; study of morphology and performance, Journal of Membrane Science, 343 (2009) 219-228.

[49] M. Safarpour, V. Vatanpour, A. Khataee, M. Esmaeili, Development of a novel high flux and fouling-resistant thin film composite nanofiltration membrane by embedding reduced graphene oxide/TiO2, Separation and Purification Technology, 154 (2015) 96-107.

[50] P. Wen, Y. Chen, X. Hu, B. Cheng, D. Liu, Y. Zhang, S. Nair, Polyamide thin film composite nanofiltration membrane modified with acyl chlorided graphene oxide, Journal of Membrane Science, 535 (2017) 208-220.

[51] S.-M. Xue, Z.-L. Xu, Y.-J. Tang, C.-H. Ji, Polypiperazine-amide nanofiltration membrane modified by different functionalized multiwalled carbon nanotubes (MWCNTs), ACS applied materials & interfaces, 8 (2016) 19135-19144.

[52] J. nan Shen, C. chao Yu, H. min Ruan, C. jie Gao, B. Van der Bruggen, Preparation and characterization of thin-film nanocomposite membranes embedded with poly (methyl methacrylate) hydrophobic modified multiwalled carbon nanotubes by interfacial polymerization, Journal of membrane science, 442 (2013) 18-26.

[53] Z. Tan, S. Chen, X. Peng, L. Zhang, C. Gao, Polyamide membranes with nanoscale Turing structures for water purification, Science, 360 (2018) 518-521.

[54] Y. Liang, Y. Zhu, C. Liu, K.-R. Lee, W.-S. Hung, Z. Wang, Y. Li, M. Elimelech, J. Jin, S. Lin, Polyamide nanofiltration membrane with highly uniform

sub-nanometre pores for sub-1 Å precision separation, Nature communications, 11 (2020) 1-9.

[55] P. Sarkar, S. Modak, S. Karan, Ultraselective and highly permeable polyamide nanofilms for ionic and molecular nanofiltration, Advanced Functional Materials, 31 (2021) 2007054.

[56] G. Gong, P. Wang, Z. Zhou, Y. Hu, New insights into the role of an interlayer for the fabrication of highly selective and permeable thin-film composite nanofiltration membrane, ACS applied materials & interfaces, 11 (2019) 7349-7356.

[57] X.L. Cao, J.L. Guo, J. Cai, M.L. Liu, S. Japip, W. Xing, S.P. Sun, The encouraging improvement of polyamide nanofiltration membrane by cucurbituril-based host–guest chemistry, AIChE Journal, 66 (2020) e16879.

[58] S. Yang, J. Wang, L. Fang, H. Lin, F. Liu, C.Y. Tang, Electrosprayed polyamide nanofiltration membrane with intercalated structure for controllable structure manipulation and enhanced separation performance, Journal of Membrane Science, 602 (2020) 117971.

[59] G. Lai, W. Lau, P. Goh, A. Ismail, N. Yusof, Y. Tan, Graphene oxide incorporated thin film nanocomposite nanofiltration membrane for enhanced salt removal performance, Desalination, 387 (2016) 14-24.

[60] X. Yang, Y. Du, X. Zhang, A. He, Z.-K. Xu, Nanofiltration membrane with a mussel-inspired interlayer for improved permeation performance, Langmuir, 33 (2017) 2318-2324.

[61] S. Zhu, S. Zhao, Z. Wang, X. Tian, M. Shi, J. Wang, S. Wang, Improved performance of polyamide thin-film composite nanofiltration membrane by using polyetersulfone/polyaniline membrane as the substrate, Journal of

Membrane Science, 493 (2015) 263-274.

[62] H. Zarrabi, M.E. Yekavalangi, V. Vatanpour, A. Shockravi, M. Safarpour, Improvement in desalination performance of thin film nanocomposite nanofiltration membrane using amine-functionalized multiwalled carbon nanotube, Desalination, 394 (2016) 83-90.

[63] J. Wang, Y. Wang, J. Zhu, Y. Zhang, J. Liu, B. Van der Bruggen, Construction of TiO2@ graphene oxide incorporated antifouling nanofiltration membrane with elevated filtration performance, Journal of Membrane Science, 533 (2017) 279-288.

[64] S.-M. Xue, C.-H. Ji, Z.-L. Xu, Y.-J. Tang, R.-H. Li, Chlorine resistant TFN nanofiltration membrane incorporated with octadecylamine-grafted GO and fluorine-containing monomer, Journal of Membrane Science, 545 (2018) 185-195.

[65] S.-L. Li, X. Shan, Y. Zhao, Y. Hu, Fabrication of a novel nanofiltration membrane with enhanced performance via interfacial polymerization through the incorporation of a new zwitterionic diamine monomer, ACS applied materials & interfaces, 11 (2019) 42846-42855.

[66] B. Zhao, Z. Guo, H. Wang, L. Wang, Y. Qian, X. Long, C. Ma, Z. Zhang, J. Li, H. Zhang, Enhanced water permeance of a polyamide thin-film composite nanofiltration membrane with a metal-organic framework interlayer, Journal of Membrane Science, 625 (2021) 119154.

[67] N. Saha, S. Joshi, Performance evaluation of thin film composite polyamide nanofiltration membrane with variation in monomer type, Journal of Membrane Science, 342 (2009) 60-69.

[68] Y. Li, Y. Su, J. Li, X. Zhao, R. Zhang, X. Fan, J. Zhu, Y. Ma, Y. Liu, Z. Jiang,

Preparation of thin film composite nanofiltration membrane with improved structural stability through the mediation of polydopamine, Journal of Membrane Science, 476 (2015) 10-19.

[69] D. Hu, Z.-L. Xu, C. Chen, Polypiperazine-amide nanofiltration membrane containing silica nanoparticles prepared by interfacial polymerization, Desalination, 301 (2012) 75-81.

[70] G. Bargeman, J. Westerink, C. Manuhutu, A. Ten Kate, The effect of membrane characteristics on nanofiltration membrane performance during processing of practically saturated salt solutions, Journal of membrane science, 485 (2015) 112-122.

[71] M. Zazouli, M. Ulbricht, S. Naseri, H. Susanto, Effect of hydrophilic and hydrophobic organic matter on amoxicillin and cephalexin residuals rejection from water by nanofiltration, (2010).

[72] N. Park, J. Cho, S. Hong, S. Lee, Ion transport characteristics in nanofiltration membranes: measurements and mechanisms, Journal of Water Supply: Research and Technology—AQUA, 59 (2010) 179-190.

[73] Y. Liu, J. Gao, Y. Ge, S. Yu, M. Liu, C. Gao, A combined interfacial polymerization and in-situ sol-gel strategy to construct composite nanofiltration membrane with improved pore size distribution and anti-protein-fouling property, Journal of Membrane Science, 623 (2021) 119097.

[74] N. Song, X. Xie, D. Chen, G. Li, H. Dong, L. Yu, L. Dong, Tailoring nanofiltration membrane with three-dimensional turing flower protuberances for water purification, Journal of Membrane Science, 621 (2021) 118985.

[75] X. Zhang, Y. Zhang, T. Wang, Z. Fan, G. Zhang, A thin film nanocomposite membrane with pre-immobilized UiO-66-NH 2 toward enhanced nanofiltration

performance, RSC advances, 9 (2019) 24802-24810.

[76] M.-B. Wu, Y. Lv, H.-C. Yang, L.-F. Liu, X. Zhang, Z.-K. Xu, Thin film composite membranes combining carbon nanotube intermediate layer and microfiltration support for high nanofiltration performances, Journal of Membrane Science, 515 (2016) 238-244.

[77] Z. Yang, Z.-w. Zhou, H. Guo, Z. Yao, X.-h. Ma, X. Song, S.-P. Feng, C.Y. Tang, Tannic acid/Fe3+ nanoscaffold for interfacial polymerization: toward enhanced nanofiltration performance, Environmental science & technology, 52 (2018) 9341-9349.
